# On the complexity of miRNA-mediated regulation in plants: novel insights into the genomic organization of plant miRNAs

**DOI:** 10.1186/1745-6150-7-15

**Published:** 2012-05-08

**Authors:** Moreno Colaiacovo, Antonella Lamontanara, Letizia Bernardo, Renzo Alberici, Cristina Crosatti, Lorenzo Giusti, Luigi Cattivelli, Primetta Faccioli

**Affiliations:** 1 CRA-Genomics Research Centre, Agricultural Research Council, via S.Protaso 302, Fiorenzuola d’Arda, PC, Italy

**Keywords:** Gene regulation, Exonic miRNA, miRNA self regulation, Plants

## Abstract

MicroRNAs (miRNAs) are endogenous small non-coding RNAs of about 20–24 nt, known to play key roles in post-transcriptional gene regulation, that can be coded either by intergenic or intragenic loci. Intragenic (exonic and intronic) miRNAs can exert a role in the transcriptional regulation and RNA processing of their host gene. Moreover, the possibility that the biogenesis of exonic miRNAs could destabilize the corresponding protein-coding transcript and reduce protein synthesis makes their characterization very intriguing and suggests a possible novel mechanism of post-transcriptional regulation of gene expression.

This work was designed to carry out the computational identification of putative exonic miRNAs in 30 plant species and the analysis of possible mechanisms involved in their regulation.

The results obtained represent a useful starting point for future studies on the complex networks involved in microRNA-mediated gene regulation in plants.

## Findings

MicroRNAs (miRNAs) are endogenous small non-coding RNAs of about 20–24 nt that can be coded either by intergenic or intragenic loci.

In plants a few examples of intronic miRNAs have been found in Arabidopsis and rice. In Arabidopsis, miR402 has been identified within the intron of gene At1g77230 in the same orientation as the hosting pre-mRNA [1]. A putative mirtron, miR1429.2, has also been identified in rice by Zhu et al. [2] as part of a homeobox gene. In Arabidopsis the intronic miR838 is particularly interesting being located in the intron of Dicer-like 1 (*Dcl1*) gene (which codes the key enzyme involved in plant miRNA maturation,[3]), and thus enabling a self-regulatory mechanism that helps maintain DCL1 homeostasis [4].

The rice miR3981 was detected in the exon of a putative glyoxalase gene and its biogenesis pathway might be involved in the regulation of glyoxalase expression. Excessive levels of glyoxalase mRNA should lead to an increase in pre-miR3981 processing by the enzyme DCL1 and with glyoxalase also being a putative target for miR3981, an increase in the amount of miRNA3981 could lead to a decrease in glyoxalase mRNA levels, thus preventing durable high expression of the gene [5]. This suggests the possibility of a balanced competition, mediated by DCL1, between glyoxalase protein production and miR3981 biogenesis leading to a fine-tuning of glyoxalase expression in rice. The possibility that the biogenesis of exonic miRNAs destabilizes the corresponding protein-coding transcript and reduces protein synthesis makes their characterization very intriguing and suggests a new mechanism of post-transcriptional regulation of gene expression.

This work was designed to carry out the computational identification of putative exonic miRNAs in plants by taking advantage of the huge amount of ESTs available for many species. The analysis of putative miRNA target sites in the host mRNA was also performed in order to gain some insight into possible novel mechanisms of gene regulation.

TCs (Tentative Consensus), from 30 plant species, publicly available from the DFCI Gene Index Project (http://compbio.dfci.harvard.edu/tgi/), were computationally screened for the presence of miRNA precursors and miRNA target sites (Additional file 1detailed methods can be found as additional file). TCs hosting miRNA precursors are reported in Additional file 2. Some of the annotated TCs refer both to a miRNA and a protein and thus represent the starting point for the identification of candidate exonic miRNAs.

The relationships between miRNAs and transcription factors (TFs) is particularly relevant, being the basis of complex regulatory networks [6,7] and several examples of TF genes hosting microRNA precursor sequence were found and are reported in aAdditional file 2: examples include AP-2, NAC and SPL coding genes. Interestingly, in some of the miRNA-containing TCs identified in this study, the miRNA host gene is also its conserved target, for example miR444, a monocot-specific miRNA identified in several monocots (barley, maize, purple false brome, rice, wheat), is part of TCs coding for MADS-box transcription factors (Additional file 2) and this family of transcription factors is also a well-known conserved target for miR444. Additional file 3 contains the example of the barley EST called GH228935 [GenBank: GH228935], which is a MADS-box-related sequence containing both the target site for miR444b (+/−) and the precursor sequence for miR444a (+/+).

All TCs or singletons that include both a miRNA precursor sequence and one or more miRNA target sites are presented in Table 1. TCs that include both a miRNA target site with +/− orientation and the miRNA precursor sequence with +/+ orientation on the coding strand can be referred to those cases where the miRNA is oriented on the same strand of the target gene. The presence of the target sequence upstream or downstream of the precursor sequence could affect both host gene post-transcriptional regulation as well as miRNA regulation or self-regulation in the case that the target site belongs to the same miRNA family as the miRNA precursor. The latter mechanism could be a further buffering system for modulation of miRNA expression and could be in accordance with the model of miRNA self-regulation proposed by Meng et al. [6,7] that suggests a feedback model in which the miRNA* binds to the complementary sites on their precursor thus exerting a cleavage-based modulating role. Examples of putative self-regulation based on the presence of a target site upstream or downstream of the precursor have been found in our data for miR444 as reported above, miR395 (rice), miR159/319 (soybean), miR1118 (wheat) and miR1219 (moss).

**Table 1:** Reports those TCs or singletons that include both a miRNA precursor sequence and one or more miRNA target sites

**Species name**	**Scientific name**	**Sequence identifier**	**Annotation from DFCI** **Gene Index**	**miRNA family**	**Target site (+/-)**	**Precursor site**
Arabidopsis	*Arabidopsis thaliana*	TC360779	pentatricopeptide (PPR) repeat-containing protein	5652	1102–1122	261-708 (+/-)
				161	1093–1113	
				400	1314–1334	

		TC383702		5636		89–166 (+/−)
				173	444–465	
Barley	*Hordeum vulgare*	GH228935	MIKC-type MADS-box transcription factor WM32A	444	246–266	280–393 (+/+)
Cotton	*Gossypum hirsutum*	TC244560		2949		366–547 (+/−)
						399–511 (+/−)
				3476	469–488	
		TC273521	Uncharacterized protein YJR115W	2949		1544–1723 (+/−)
						1576–1689 (+/−)
				3476	1647–1666	
Maize	*Zea mays*	TC498468	Transcriptional regulator, TetR family	168		42–145 (+/+)
				396	814–833	
Moss	*Physcomitrella patens*	NP13127877		1219	535–555	263–414 (+/−)
Medicago truncatula	*Medicago truncatula*	EX529600		2629		171–231 (+/−)
				2627	173–194	
		NP7251801		5241		1186–1260 (+/+)
				1510	674–694	
				5242	1341–1361	
				2678	86–106	
		NP7252398		5241		1163–1280 (+/+)
						1186–1260 (+/+)
						1189–1255 (+/+)
				1510	674–694	
				5242	1341–1361	
				2678	86–106	
		NP7252404		5241		1189–1255 (+/+)
				1510	674–694	
				2678	86–106	
		NP7254391		2674		467–760 (+/+)
				2089	200–221	
Moss	*Physcomitrella patens*	TC19973	RNA polymerase II largest subunit	899		323–544 (+/+)
				2083	416–436	
					217–236	
		TC24438	Predicted protein	1062		378–561 (+/+)
				529	581–600	
		TC27593	Ribulose bisphosphate carboxylase small chain	899		48–269 (+/–)
				2083	354–374	
					154–174	
Rice	*Oryza sativa*	CB677501	Os05g0439200 protein	156/157		219–338 (+/+)
						252–352 (+/+)
				168	217–236	
		CX099912		156		187–351 (+/+)
						220–320 (+/+)
				168	205–224	
		EG709456	Conserved protein	2118		109–285 (+/+)
				1869	239–259	
		CA760441		395	539–559	373–441 (+/−)
						230–397 (+/−)
						87–154 (+/−)
Soybean	*Glycine max*	BW657280	Transcriptional regulator, MarR family	4414		164–291 (+/+)
				159/319	123–144	
		TC425684	Chromosome chr14 scaffold_190, whole genome shotgun sequence	4409		479–539 (+/+)
				5372		346–410 (+/+)
				5674	373–393	
		TC428562	Transcriptional regulator, MarR family	4414		563–690 (+/+)
				319	522–543	
		TC442024	MATE efflux family protein	159/319	324–343	358–460 (+/+)
		TC443643		5668		717–829 (+/−)
				1510	697–717	
		TC451566		5038		424–574 (+/+)
						482–556 (+/+)
				5670	567–586	
Spruce	*Picea abies*	EX343185		3700		281–358 (+/+)
				947	215–236	
		EX369017		3700		217–294 (+/+)
				947	151–172	
Wheat	*Triticum aestivum*	TC369361	Calmodulin TaCaM2-1	1118	727–749	918–1057 (+/+)
		TC370181	Calmodulin TaCaM2-3	1118	644–666	835–974 (+/+)

On the other hand, those TCs where the orientation of miRNA precursor sequence with respect to coding strand is +/− should refer to those miRNAs coded in the antisense orientation with respect to the host gene. Antisense transcription associated with miRNA target mRNAs have been previously discovered in Arabidopsis [8] and may serve as a link between miRNA and RNA silencing pathways, suggesting that miRNAs may have additional roles in post-transcriptional regulation that are independent of cleavage.

TCs or singletons coding for more than one miRNA precursor (miRNA clusters) have also been identified both in the +/+ and +/− orientations (sense/antisense miRNAs) and are summarized in Additional file 4. The occurrence of pairs of antisense miRNAs in plants has been previously reported [9]. Most of the miRNA clusters highlighted in plants contain several members of a specific miRNA family rather than encoding miRNA with unrelated sequences, as is frequently the case for animal miRNA clusters [10]. Examples of plant miRNA clusters with members of different families are reported in Additional file 3.

Among the exonic miRNAs identified, we chose to explore, as an interesting example, the relationship between miR1118 and calmodulin-coding genes in wheat (Table 1). Calmodulin genes are of particular interest due to the fundamental role of calmodulin in the calcium-dependent regulation during plant response to endogenous and exogenous stimuli [11]. Calmodulin-binding proteins have already been shown to be targeted by miRNAs in plants [12], while no report is available on miRNA targeting to calmodulin itself.

Many plant species possess calmodulin (CaM) multigene families composed of several genes: some of which encode identical proteins, while others encode different isoforms of calmodulin-related proteins. In wheat, at least 13 CaM-related genes have been identified and classified into four subfamilies (SF-1:4 on the basis of their nucleotide sequence) coding for three distinct isoforms named TaCaM-I, TaCaM-II and TaCaM-III [13]. Genes from SF-1, SF-3 and SF-4 encode for the same isoform (TaCaM-I, the most similar to calmodulin from other plant species and organisms), while SF-2, the most divergent calmodulin subfamily with most recent origin in wheat, encodes at least two isoforms (TaCaM-II and TaCaM-III).

**Figure 1:**
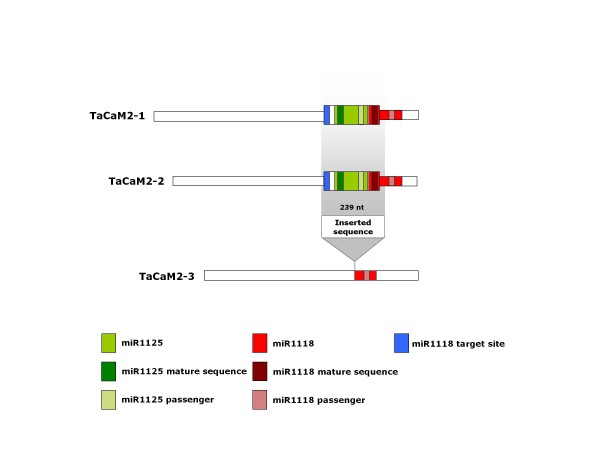
**Reports the positions of miR1118 and miR1125 coding sequences inside wheat SF-2 calmodulin genes (*TaCaM2-1*,*TaCaM2-2*,*TaCaM2-3*).**

To deeply characterize the calmodulin gene family for the presence of miRNA-coding sequences, ten cDNA clones corresponding to 10 different genes encoding calmodulin-like proteins were screened against the wheat miRNA mature sequences present in miRBase. Each cDNA clone contained at least part of the 5′UTR, the full coding region, the 3′-UTR and the polyA tail. Blast output is reported in Additional file 5. The most significant match was found for tae-miR1118 mature sequence (score 43, e-value 2e-006), furthermore, an additional miRNA (tae-miR1125 mature sequence) showed a very significant match (score 39; e-value 2e-005). Both miRNAs were found in the two genes belonging to SF-2 (*TaCaM2-1* and *TaCaM2-2*), but not in the third sequence belonging to the same subfamily (*TaCaM2-3*). Mature sequences for both miRNAs are included in the 3′UTR regions of the two calmodulin genes. Although the coding sequences of *TaCaM2-1* and *TaCaM2-2* are substantially different and produce two distinguishable calmodulin isoforms (TaCaM-III and TaCaM-II, respectively), the corresponding 3′UTR regions differ only by one nucleotide. The nucleotide sequence alignment between the 3′ UTR regions of the three calmodulin genes belonging to SF-2 revealed a striking difference between *TaCaM2-3* and the other two genes: *TaCaM2-1* and *TaCaM2-2* share a long insertion of 239 bp that is absent in *TaCaM2-3* (Figure 1 and Additional file 6). A Blast search against the complete TREP database, containing a collection of repetitive DNA sequences from different *Triticeae* species, highlighted a strong match (score 315, e-value 1e-86) of the inserted sequence with a DNA transposon of the Mariner class called DTT_Polyphemus_464G14-3. This finding is in agreement with a possible model for the evolution of MIR genes that proposes their origin from transposable elements, on the basis of the observation that the DNA-type nonautonomous elements called MITE (miniature inverted-repeat transposable element) can fold into imperfect stem loops that are typical of miRNA precursors [3]. Several pieces of evidence pointed to plant *mariner*-like elements (MLEs) as the autonomous partners of the nonautonomous *Stowaway* MITEs [14].

The presence of the transposon-related insertion in calmodulin genes from other plant species has was investigated, and a blast search using the sequence of *TaCaM2-2* gene as query was performed. The results are reported in Additional file 7: a significant match with the 3′UTR region of the wheat gene was detected only with a barley gene, where, however, the transposon-related sequence was absent.

Moreover, while the miR1125 precursor sequence deposited in the miRBase found a significant match (81% identity) entirely included in the inserted sequence, the miR1118 precursors sequence matched (100% identity) only partially into the insertion while it spanned also the very last part of the 3′UTR of the *TaCaM* genes (Figure 1). The reverse complement sequence for miR1118 found inside the 239 nt insertion could thus represent a target site for the same miRNA. This is very possible since only 3 mismatches were found when compared with the mature sequence, and the target site is also predicted by psRNAtarget. A second reverse complement site is also shared by all three SF-2 genes (*TaCaM2-1*, *TaCaM2-2* and *TaCaM2-3*), being located in the last part of the 3′ UTR region. This site corresponds to the miRNA* of the precursor deposited in the miRBase and it can be considered a less likely target site due to the high number of mismatches with the mature sequence. However, the prediction of the RNA secondary structures by mFold software (Additional file 8) highlighted the worse energetic stability of the miR1118 precursor stored in miRBase compared to that of the inserted sequence (−32.5 kcal/mol vs.−117.6 kcal/mol). Although the transposable element is longer than the deposited precursor (239 nt vs. 140 nt) it has a Minimal Folding Energy Index (MFEI) higher than the miRNA precursor (1.16 vs. 0.59) and it is in better agreement with the values known for plant miRNAs. The existence of two alternative miR1118 precursors could thus be possible. The inserted sequence has acquired some mutations since the insertion event and these variations have led to a better stability of the candidate miRNA precursor, thus confirming this hypothesis. In fact, compared with the inserted sequence, the predicted secondary structure of the transposable element stored in the TREP database shows a worse stability (−84.4 kcal/mol), a lower MFEI (0.88) and a higher number of mismatches (4) between miRNA and miRNA*.

## Conclusions

Recently Meng et al. [6,7] reported on the high dynamicity of the regulatory activities mediated by miRNAs which are themselves strictly regulated, thus making the miRNA-involved networks more robust.

The results presented in this work can be considered as a valuable support for future studies on the complex networks involved in miRNA-mediated gene regulation in plants. In particular the relationships between miRNAs and transcription factors (TFs) represent a key node of these complex networks. Most of the currently available studies have been involved in the identification of TF recognition sites in the promoter of miRNA genes and in the identification of miRNA target sites in TF coding genes. The present work adds a novel level of complexity pointing out the possibility that miRNA coding sequences can also be hosted by TF genes.

## **Open peer review**

Reviewed by Dr Alexander Max Burroughs (nominated by Dr L Aravind) and Dr Raya Khanin (nominated by Arcady Mushegian). For the full reviews, please go to the Reviewers’ comments section.

**Reviewers:** This article was reviewed by Dr Alexander Max Burroughs (nominated by Dr L Aravind) and Dr Raya Khanin (nominated by Arcady Mushegian).

## Competing interests

The authors declare that they have no competing interests.

## Authors’ contribution

PF conceived the idea and wrote the paper. MC and AL did most of the computational work and were supported by LB. RA gave the informatics support. LC, CC, LG contributed to the interpretation of the results and to the preparation of manuscript. All authors read and approved the final manuscript.

## **Reviewers’ comments**

## ***Reviewer’s report 1***

Dr Alexander Max Burroughs (nominated by Dr L Aravind)

The authors probe TCs and identify several miRNA precursors. What is missing from this analysis is a sense of the novelty of the findings. How many of these precursors have previously been predicted as being present at in the genome at the identified locations? For reasonably well-annotated genomes like Arabidopsis or rice it should be relatively easy to look at this to provide an overview of how many times novel locations are identified for known miRNAs. If this count is large, it may be interesting to discuss why these have not been identified in the past.

**Author response:***In order to clarify this aspect, we checked all the miRNA genomic locations as they are reported in miRBase, and we compared them with the location of the identified TCs. We performed the analysis only for Arabidopsis sequences, which are supposed to be better annotated. Almost all pairs miRNA-TC show overlapping locations, meaning that the two pieces of information were previously known to the scientific community. However, the novelty of our work is that we merged these existing data and we were able to extract novel findings which are relevant for the miRNA analysis, e.g. the characterization of these miRNA precursors as exonic miRNAs.*

As an additional form of evidence it might be useful, when available, to map deep-sequencing data to the TCs in order to confirm the presence and active processing of the identified precursors, particularly any identified in novel locations. In addition, one limitation in the analysis is that it precludes detection of previously unidentified miRNA precursors, an angle which could be addressed by feeding the deep-sequencing data mappings into a miRNA prediction program (based on parameters like mature/star sequence count distribution, folding properties, etc.) like miRanalyzer or miRdeep. This could increase the number of transcripts potentially harboring exonic miRNA precursors.

**Author response:***Small RNA sequencing is an interesting further development for this work and we plan to have new data in the near future which will be part of another publication. At the moment we are in the process of sequencing small RNAs from several plant species, and this will give us the opportunity to study also novel microRNAs.*

I was struck by the general lack of conservation in the locations of the identified precursors: few seemed to be located in homologous transcripts across different plant species. While the authors discuss an example of conservation of the miR444 precursor, it might be informative to give an overview of the general conservation/lack of conservation observed in the localization of precursors in host transcripts. Are transcripts harboring both the precursor and the miRNA target sequence more likely to be conserved? This could provide some general insight into the mobility of the precursors and their role in “cis-regulation” of transcripts.

**Author response:***According to our analysis, there are few miRNA precursors which are present in conserved transcripts across different species. Of all the miRNA families which were identified in multiple species, approximately 20% show host conservation in at least two species. The situation is similar if we consider transcripts harboring both the precursor and the target site. A possible explanation for this finding could be the different level of completeness of species-specific gene indices (e.g. accuracy of TC annotations).*

The authors note “On the other hand, those TCs where the orientation of miRNA precursor sequence with respect to coding strand is +/− should refer to those miRNAs coded in the antisense orientation with respect to the host gene. Antisense transcription associated with miRNA target mRNAs have been previously discovered in Arabidopsis and may serve as a link between miRNA and RNA silencing pathways…” This is a very interesting observation and quite consistent with a growing number of studies linking antisense transcription at gene loci to RNAi pathways. It might be worthwhile to scan existing gene annotations for evidence of downstream, noncoding antisense transcription which could overlap the gene body in +/− cases; this could provide some global evidence in support of the statement.

**Author response:***We manually inspected all the +/− cases which we identified in Arabidopsis (chosen for the same reasons reported above, i.e. the accuracy of genome annotation), then exploring the TAIR genome browser and looking for evidences of antisense transcription at those locations. We found evidence for antisense transcription in 38% of the cases.*

Outside of the analysis of the calmodulin transcripts, there was little discussion of the predicted folding energies for the identified precursors. It could be instructive to see how secondary structure stabilities in the identified precursor compare to other precursors in individual genomes, foremost as a gauge of the reliability of the identified precursors, particularly those precursors identified in novel locations (see first comment).

**Author response:***The criteria which we used for the prediction of miRNA precursors are very stringent, since we selected as putative miRNAs only those sequences which have a nucleotide identity greater than 95% with a known precursor. Because of this, we don’t expect big changes in the energetic stability of the identified sequences compared to the ones stored in miRBase. The variations are supposed to be minimal and should not affect dramatically the miRNA stability. However, in order to confirm our expectation, we selected some miRNAs at random and we compared their energy values to the corresponding sequence stored in miRBase, by using the mFold software. The differences are small: ath-MIR416 has a free energy equal to −27.9 kcal/mol if we consider the miRBase sequence, and −31 kcal/mol if we consider our sequence; the same is true also for ath-MIR398b (−51.5 vs −51.4), ath-MIR319b (−81.3 vs −80.4) and osa-MIR439d (−57.8 vs −60.8).*

## ***Reviewer’s report 2***

Dr Raya Khanin (nominated by Arcady Mushegian)

Results are given as flat files. The paper would benefit from organizing the results in a searchable database.

**Author response:***We are planning to set up a searchable database with all the relevant information.*

## Supplementary Material

Additional file 1 Reports the results of blasting each species-specific Gene Index against the corresponding species-specific miRNA precursor sequences. Click here for file

Additional file 2 Reports barley EST named GH228935 which includes both the target site for miR444b (+/−) and the precursor sequence for miR444a (+/+). Click here for file

Additional file 3 Reports TCs or singletons coding for more than one miRNA precursor (miRNA clusters) both in the +/+ and +/− orientations (sense/antisense miRNAs). Click here for file

Additional file 4 Blast results for ten wheat cDNA clones (corresponding to 10 different genes encoding calmodulin-like proteins) against the wheat miRNA mature sequences present in miRBase. Click here for file

Additional file 5 **Sequences of wheat SF-2 calmodulin genes (*TaCaM2-1*,*TaCaM2-2*,*TaCaM2-3*).** Precursors, mature and reverse complement sequences are reported for miR1118 and miR1125. Click here for file

Additional file 6 **Blast results related to the presence of the transposon-related insertion in calmodulin genes from plant species other than wheat using the sequence of*TaCaM2-2*gene as query.** Click here for file

Additional file 7 Reports the predicted secondary structures of the three miRNA precursors putatively coded by the calmodulin mRNA: the miR1118 precursor identical to the one present in miRBase, the miR1118 and miR1225 precursors putatively coded by the transposon insertion. Click here for file

Additional file 8 **Reports detailed on the adopted computational methods**[15-17]. Click here for file
