# Supplementary material for: On the complexity of miRNA-mediated regulation in plants: novel insights into the genomic organization of plant miRNAs
Source: Biol Direct. 2012 May 8;7:15. doi: 10.1186/1745-6150-7-15 (PMC3464803; doi:10.1186/1745-6150-7-15)
Supplement: Additional file 2 — Reports barley EST named GH228935 which includes both the target site for miR444b (+/−) and the precursor sequence for miR444a (+/+). [file 1745-6150-7-15-S2.doc]

>HVSMEn0024P10r2 GH228935 HV__Ea0048D04f

CACCGCGTCGTCATTCCCCTCCCCGCCTTTTTTTCTATCCCCCCACTTGCGGCTTCTGGATCGGCCCCGATCCTTCCAGACACTTCCGCCCCCCCGCTATAAAAGGCCGGCGAGCGCCACCAGACCCGCCATCAGCGCTCGCTCGCACGCACCAACCCAACCCAACCCAACCCTACCCTACTCCGTTCGCGCCCGGTTGGTGGCTGGAGCCCGCGGAGGACTCGAGATACGCATGTGGCGGCACC**AAGCATGAGGCAACAACTGCA**GTACTTGTGGGGAAGGCACAAGTAGGACACCAACATTACCTGCAAGCAAGACGCAAAATTAATAGAACATCACCATACTTGTGGCTTTCTTGCAAGTCGTGCAGTTGCTGCCTCAAGCTTGCTGCCTCCCTTTGCCAAAGCTATCAGAAAAAAACATAAAGTTGTGTTAGTTTCCCTAAACTGTAACACATGTTTTACAAATTGATCTAGAAATGATTGGCTCACTGTTAATTTAAACCAATAATTACTCGATAACTGCATATGCACATGTTATGGAAGTAAGGCGGTAGAATACTTATGTTTTCATCTACTGGCAGGTAGCAACACGTATAGGTATATCACTCTGA

**miR444b target sequence (+/-)**

miR444a precursor sequence (+/+)
