# Supplementary material for: On the complexity of miRNA-mediated regulation in plants: novel insights into the genomic organization of plant miRNAs
Source: Biol Direct. 2012 May 8;7:15. doi: 10.1186/1745-6150-7-15 (PMC3464803; doi:10.1186/1745-6150-7-15)
Supplement: Additional file 3 — Reports TCs or singletons coding for more than one miRNA precursor (miRNA clusters) both in the +/+ and +/− orientations (sense/antisense miRNAs). [file 1745-6150-7-15-S3.doc]

| Species name | TC | Annotation as from DFCI Gene Index | miRNA | Precursor site | orientation |
| --- | --- | --- | --- | --- | --- |
| *Medicago truncatula* | AW584548 | Uncharacterized protein MG242 | miR398 | 29-126 | +/+ |
|  |  |  | miR2119 | 304-433 | +/+ |
|  | TC172457 | RNA-directed DNA polymerase (Reverse transcriptase) | miR2611 | 4199-4495 | +/+ |
|  |  |  | miR2680 | 3099-3266 | +/- |
|  | TC174428 | Cyclin-like F-box | miR5260 | 277-374 | +/- |
|  |  |  | miR5215 | 459-544 | +/- |
|  | TC183456 |  | miR398 | 28-125 | +/+ |
|  |  |  | miR2119 | 303-432 | +/+ |
|  | TC188466 | NADH dehydrogenase subunit 4 | miR5260 | 328-425 | +/- |
|  |  |  | miR5215 | 510-595 | +/- |
| Rice | CR279647 | WD40-like beta propeller repeat protein | miR1318 | 164-322 | +/+ |
|  |  |  | miR1432 | 193-301 | +/+ |
|  | TC506707 |  | miR1317 | 1271-1431 | +/- |
|  |  |  | miR1882 | 1282-1422 | +/- |
|  |  |  | miR1882 | 1286-1417 | +/+ |
|  | TC534044 |  | miR1318 | 133-291 | +/+ |
|  |  |  | miR1432 | 162-270 | +/- |
| Spruce | TC136824 |  | miR482 | 144-261 | +/+ |
|  |  |  | miR3704 | 347-464 | +/- |
|  | TC160192 | Resistance protein PLTR | miR3704 | 252-369 | +/- |
|  |  |  | miR482 | 49-166 | +/+ |
|  | TC162739 | MGC115225 protein | miR482 | 53-170 | +/+ |
|  |  |  | miR3704 | 256-373 | +/- |
| Poplar | TC150962 |  | miR482 | 44-150 | +/+ |
|  |  |  | miR1448 | 231-315 | +/+ |
|  | TC151228 | SJCHGC02105 protein | miR482 | 31-137 | +/+ |
|  |  |  | miR1448 | 218-302 | +/+ |
|  | TC161498 | Chromosome chr13 scaffold_48, whole genome shotgun sequence | miR482 | 20-126 | +/+ |
|  |  |  | miR1448 | 207-291 | +/+ |
|  | TC164054 | Voltage-dependent calcium channel gamma-like subunit (Neuronal voltage-gated calcium channel gamma-like subunit) (Transmembrane protein 37). | miR482 | 75-181 | +/+ |
|  |  |  | miR1448 | 262-346 | +/+ |
|  | TC171122 |  | miR171 | 65-176 | +/+ |
|  |  |  | miR479 | 79-163 | +/+ |
| Tall fescue | TC16625 | Squamosa promoter-binding-like protein 14 | miR156 | 402-581 | +/- |
|  |  |  | miR529 | 456-572 | +/- |
| Pine | TC189235 |  | miR159 | 1133-1342 | +/- |
|  |  |  | miR319 | 1133-1338 | +/+ |
| Moss | TC19973 | RNA polymerase II largest subunit | miR2083 | 142-462 | +/+ |
|  |  |  | miR899 | 323-544 | +/+ |
|  |  |  |  |  |  |
