# Supplementary material for: On the complexity of miRNA-mediated regulation in plants: novel insights into the genomic organization of plant miRNAs
Source: Biol Direct. 2012 May 8;7:15. doi: 10.1186/1745-6150-7-15 (PMC3464803; doi:10.1186/1745-6150-7-15)
Supplement: Additional file 4 — Blast results for ten wheat cDNA clones (corresponding to 10 different genes encoding calmodulin-like proteins) against the wheat miRNA mature sequences present in miRBase. [file 1745-6150-7-15-S4.doc]

BLASTN 2.2.21 [Jun-14-2009]

Reference: Altschul, Stephen F., Thomas L. Madden, Alejandro A. Schaffer,

Jinghui Zhang, Zheng Zhang, Webb Miller, and David J. Lipman (1997),

"Gapped BLAST and PSI-BLAST: a new generation of protein database search

programs", Nucleic Acids Res. 25:3389-3402.

Query= gi|1754990|gb|U48242.1|TAU48242 Triticum aestivum calmodulin

TaCaM1-1 mRNA, complete cds

(816 letters)

Database: matureREL16.txt

4142 sequences; 88,243 total letters

Searching..................................................done

Score E

Sequences producing significant alignments: (bits) Value

smo-miR1100 MIMAT0005256 23 1.8

tae-miR167b MIMAT0018214 21 6.2

tae-miR1130 MIMAT0005365 21 6.2

osa-miR1320-3p MIMAT0015286 21 6.2

osa-miR2091-5p MIMAT0010046 21 6.2

osa-miR1320-3p MIMAT0015286 21 6.2

osa-miR2091-5p MIMAT0010046 21 6.2

ptc-miR1449 MIMAT0006011 21 6.2

aqc-miR408 MIMAT0012581 21 6.2

mtr-miR2620 MIMAT0013340 21 6.2

gma-miR4393b MIMAT0018317 21 6.2

ath-miR1886.3 MIMAT0013773 21 6.2

aly-miR163.2 MIMAT0017679 21 6.2

aly-miR3445.1* MIMAT0017724 21 6.2

aly-miR390b* MIMAT0017536 21 6.2

aly-miR3433* MIMAT0017685 21 6.2

ppt-miR1059 MIMAT0005177 21 6.2

>smo-miR1100 MIMAT0005256

Length = 22

Score = 22.9 bits (24), Expect = 1.8

Identities = 14/15 (93%)

Strand = Plus / Plus

Query: 245 gggcagaaccccact 259

|| ||||||||||||

Sbjct: 7 ggacagaaccccact 21

>tae-miR167b MIMAT0018214

Length = 21

Score = 21.1 bits (22), Expect = 6.2

Identities = 11/11 (100%)

Strand = Plus / Plus

Query: 469 gaagctgacag 479

|||||||||||

Sbjct: 2 gaagctgacag 12

>tae-miR1130 MIMAT0005365

Length = 23

Score = 21.1 bits (22), Expect = 6.2

Identities = 11/11 (100%)

Strand = Plus / Plus

Query: 92 cctccgtctcg 102

|||||||||||

Sbjct: 1 cctccgtctcg 11

>osa-miR1320-3p MIMAT0015286

Length = 21

Score = 21.1 bits (22), Expect = 6.2

Identities = 14/16 (87%)

Strand = Plus / Minus

Query: 307 tggaacgattgacttt 322

|||||||| ||| |||

Sbjct: 20 tggaacgaatgaattt 5

>osa-miR2091-5p MIMAT0010046

Length = 21

Score = 21.1 bits (22), Expect = 6.2

Identities = 11/11 (100%)

Strand = Plus / Plus

Query: 369 ccgaggaggag 379

|||||||||||

Sbjct: 10 ccgaggaggag 20

Score = 21.1 bits (22), Expect = 6.2

Identities = 14/16 (87%)

Strand = Plus / Minus

Query: 307 tggaacgattgacttt 322

|||||||| ||| |||

Sbjct: 20 tggaacgaatgaattt 5

Score = 21.1 bits (22), Expect = 6.2

Identities = 11/11 (100%)

Strand = Plus / Plus

Query: 369 ccgaggaggag 379

|||||||||||

Sbjct: 10 ccgaggaggag 20

>ptc-miR1449 MIMAT0006011

Length = 22

Score = 21.1 bits (22), Expect = 6.2

Identities = 11/11 (100%)

Strand = Plus / Plus

Query: 344 gcacgtaagat 354

|||||||||||

Sbjct: 7 gcacgtaagat 17

>aqc-miR408 MIMAT0012581

Length = 21

Score = 21.1 bits (22), Expect = 6.2

Identities = 11/11 (100%)

Strand = Plus / Minus

Query: 259 tgaggcagagc 269

|||||||||||

Sbjct: 12 tgaggcagagc 2

>mtr-miR2620 MIMAT0013340

Length = 22

Score = 21.1 bits (22), Expect = 6.2

Identities = 14/16 (87%)

Strand = Plus / Plus

Query: 354 tgaaagacaccgactc 369

||| |||||||| |||

Sbjct: 4 tgatagacaccggctc 19

>gma-miR4393b MIMAT0018317

Length = 24

Score = 21.1 bits (22), Expect = 6.2

Identities = 14/16 (87%)

Strand = Plus / Plus

Query: 624 ttgaaaactgacagca 639

||||||| |||||||

Sbjct: 1 ttgaaaagggacagca 16

>ath-miR1886.3 MIMAT0013773

Length = 21

Score = 21.1 bits (22), Expect = 6.2

Identities = 11/11 (100%)

Strand = Plus / Minus

Query: 348 gtaagatgaaa 358

|||||||||||

Sbjct: 20 gtaagatgaaa 10

>aly-miR163.2 MIMAT0017679

Length = 21

Score = 21.1 bits (22), Expect = 6.2

Identities = 13/14 (92%)

Strand = Plus / Plus

Query: 505 tgaggctgatgtcg 518

||||||||| ||||

Sbjct: 7 tgaggctgaagtcg 20

>aly-miR3445.1* MIMAT0017724

Length = 21

Score = 21.1 bits (22), Expect = 6.2

Identities = 15/16 (93%), Gaps = 1/16 (6%)

Strand = Plus / Minus

Query: 547 gtttgtgaaggtgatg 562

||||| ||||||||||

Sbjct: 20 gtttg-gaaggtgatg 6

>aly-miR390b* MIMAT0017536

Length = 21

Score = 21.1 bits (22), Expect = 6.2

Identities = 11/11 (100%)

Strand = Plus / Plus

Query: 322 tcctgagttcc 332

|||||||||||

Sbjct: 10 tcctgagttcc 20

>aly-miR3433* MIMAT0017685

Length = 21

Score = 21.1 bits (22), Expect = 6.2

Identities = 11/11 (100%)

Strand = Plus / Plus

Query: 225 gaactgtgatg 235

|||||||||||

Sbjct: 11 gaactgtgatg 21

>ppt-miR1059 MIMAT0005177

Length = 21

Score = 21.1 bits (22), Expect = 6.2

Identities = 11/11 (100%)

Strand = Plus / Minus

Query: 547 gtttgtgaagg 557

|||||||||||

Sbjct: 18 gtttgtgaagg 8

BLASTN 2.2.21 [Jun-14-2009]

Reference: Altschul, Stephen F., Thomas L. Madden, Alejandro A. Schaffer,

Jinghui Zhang, Zheng Zhang, Webb Miller, and David J. Lipman (1997),

"Gapped BLAST and PSI-BLAST: a new generation of protein database search

programs", Nucleic Acids Res. 25:3389-3402.

Query= gi|1754992|gb|U48688.1|TAU48688 Triticum aestivum calmodulin

TaCaM1-2 mRNA, complete cds

(796 letters)

Database: matureREL16.txt

4142 sequences; 88,243 total letters

Searching..................................................done

Score E

Sequences producing significant alignments: (bits) Value

smo-miR1100 MIMAT0005256 23 1.7

tae-miR167b MIMAT0018214 21 6.1

tae-miR1130 MIMAT0005365 21 6.1

osa-miR1320-3p MIMAT0015286 21 6.1

osa-miR2091-5p MIMAT0010046 21 6.1

osa-miR1320-3p MIMAT0015286 21 6.1

osa-miR2091-5p MIMAT0010046 21 6.1

ptc-miR1449 MIMAT0006011 21 6.1

aqc-miR408 MIMAT0012581 21 6.1

mtr-miR2620 MIMAT0013340 21 6.1

gma-miR4393b MIMAT0018317 21 6.1

ath-miR1886.3 MIMAT0013773 21 6.1

aly-miR163.2 MIMAT0017679 21 6.1

aly-miR3445.1* MIMAT0017724 21 6.1

aly-miR390b* MIMAT0017536 21 6.1

aly-miR3433* MIMAT0017685 21 6.1

ppt-miR1059 MIMAT0005177 21 6.1

>smo-miR1100 MIMAT0005256

Length = 22

Score = 22.9 bits (24), Expect = 1.7

Identities = 14/15 (93%)

Strand = Plus / Plus

Query: 249 gggcagaaccccact 263

|| ||||||||||||

Sbjct: 7 ggacagaaccccact 21

>tae-miR167b MIMAT0018214

Length = 21

Score = 21.1 bits (22), Expect = 6.1

Identities = 11/11 (100%)

Strand = Plus / Plus

Query: 473 gaagctgacag 483

|||||||||||

Sbjct: 2 gaagctgacag 12

>tae-miR1130 MIMAT0005365

Length = 23

Score = 21.1 bits (22), Expect = 6.1

Identities = 11/11 (100%)

Strand = Plus / Plus

Query: 96 cctccgtctcg 106

|||||||||||

Sbjct: 1 cctccgtctcg 11

>osa-miR1320-3p MIMAT0015286

Length = 21

Score = 21.1 bits (22), Expect = 6.1

Identities = 14/16 (87%)

Strand = Plus / Minus

Query: 311 tggaacgattgacttt 326

|||||||| ||| |||

Sbjct: 20 tggaacgaatgaattt 5

>osa-miR2091-5p MIMAT0010046

Length = 21

Score = 21.1 bits (22), Expect = 6.1

Identities = 11/11 (100%)

Strand = Plus / Plus

Query: 373 ccgaggaggag 383

|||||||||||

Sbjct: 10 ccgaggaggag 20

Score = 21.1 bits (22), Expect = 6.1

Identities = 14/16 (87%)

Strand = Plus / Minus

Query: 311 tggaacgattgacttt 326

|||||||| ||| |||

Sbjct: 20 tggaacgaatgaattt 5

Score = 21.1 bits (22), Expect = 6.1

Identities = 11/11 (100%)

Strand = Plus / Plus

Query: 373 ccgaggaggag 383

|||||||||||

Sbjct: 10 ccgaggaggag 20

>ptc-miR1449 MIMAT0006011

Length = 22

Score = 21.1 bits (22), Expect = 6.1

Identities = 11/11 (100%)

Strand = Plus / Plus

Query: 348 gcacgtaagat 358

|||||||||||

Sbjct: 7 gcacgtaagat 17

>aqc-miR408 MIMAT0012581

Length = 21

Score = 21.1 bits (22), Expect = 6.1

Identities = 11/11 (100%)

Strand = Plus / Minus

Query: 263 tgaggcagagc 273

|||||||||||

Sbjct: 12 tgaggcagagc 2

>mtr-miR2620 MIMAT0013340

Length = 22

Score = 21.1 bits (22), Expect = 6.1

Identities = 14/16 (87%)

Strand = Plus / Plus

Query: 358 tgaaagacaccgactc 373

||| |||||||| |||

Sbjct: 4 tgatagacaccggctc 19

>gma-miR4393b MIMAT0018317

Length = 24

Score = 21.1 bits (22), Expect = 6.1

Identities = 14/16 (87%)

Strand = Plus / Plus

Query: 628 ttgaaaactgacagca 643

||||||| |||||||

Sbjct: 1 ttgaaaagggacagca 16

>ath-miR1886.3 MIMAT0013773

Length = 21

Score = 21.1 bits (22), Expect = 6.1

Identities = 11/11 (100%)

Strand = Plus / Minus

Query: 352 gtaagatgaaa 362

|||||||||||

Sbjct: 20 gtaagatgaaa 10

>aly-miR163.2 MIMAT0017679

Length = 21

Score = 21.1 bits (22), Expect = 6.1

Identities = 13/14 (92%)

Strand = Plus / Plus

Query: 509 tgaggctgatgtcg 522

||||||||| ||||

Sbjct: 7 tgaggctgaagtcg 20

>aly-miR3445.1* MIMAT0017724

Length = 21

Score = 21.1 bits (22), Expect = 6.1

Identities = 15/16 (93%), Gaps = 1/16 (6%)

Strand = Plus / Minus

Query: 551 gtttgtgaaggtgatg 566

||||| ||||||||||

Sbjct: 20 gtttg-gaaggtgatg 6

>aly-miR390b* MIMAT0017536

Length = 21

Score = 21.1 bits (22), Expect = 6.1

Identities = 11/11 (100%)

Strand = Plus / Plus

Query: 326 tcctgagttcc 336

|||||||||||

Sbjct: 10 tcctgagttcc 20

>aly-miR3433* MIMAT0017685

Length = 21

Score = 21.1 bits (22), Expect = 6.1

Identities = 11/11 (100%)

Strand = Plus / Plus

Query: 229 gaactgtgatg 239

|||||||||||

Sbjct: 11 gaactgtgatg 21

>ppt-miR1059 MIMAT0005177

Length = 21

Score = 21.1 bits (22), Expect = 6.1

Identities = 11/11 (100%)

Strand = Plus / Minus

Query: 551 gtttgtgaagg 561

|||||||||||

Sbjct: 18 gtttgtgaagg 8

BLASTN 2.2.21 [Jun-14-2009]

Reference: Altschul, Stephen F., Thomas L. Madden, Alejandro A. Schaffer,

Jinghui Zhang, Zheng Zhang, Webb Miller, and David J. Lipman (1997),

"Gapped BLAST and PSI-BLAST: a new generation of protein database search

programs", Nucleic Acids Res. 25:3389-3402.

Query= gi|1754994|gb|U48689.1|TAU48689 Triticum aestivum calmodulin

TaCaM1-3 mRNA, complete cds

(779 letters)

Database: matureREL16.txt

4142 sequences; 88,243 total letters

Searching..................................................done

Score E

Sequences producing significant alignments: (bits) Value

aly-miR4241 MIMAT0017930 23 1.7

smo-miR1100 MIMAT0005256 23 1.7

zma-miR395l* MIMAT0015344 21 5.9

tae-miR167b MIMAT0018214 21 5.9

osa-miR1320-3p MIMAT0015286 21 5.9

osa-miR2091-5p MIMAT0010046 21 5.9

osa-miR1320-3p MIMAT0015286 21 5.9

osa-miR2091-5p MIMAT0010046 21 5.9

ptc-miR1449 MIMAT0006011 21 5.9

aqc-miR408 MIMAT0012581 21 5.9

gma-miR4393b MIMAT0018317 21 5.9

aly-miR3445.1* MIMAT0017724 21 5.9

aly-miR390b* MIMAT0017536 21 5.9

aly-miR3433* MIMAT0017685 21 5.9

ppt-miR1059 MIMAT0005177 21 5.9

>aly-miR4241 MIMAT0017930

Length = 22

Score = 22.9 bits (24), Expect = 1.7

Identities = 15/17 (88%)

Strand = Plus / Plus

Query: 639 tttggattttcttgcag 655

|| |||||||||| |||

Sbjct: 3 ttgggattttcttacag 19

>smo-miR1100 MIMAT0005256

Length = 22

Score = 22.9 bits (24), Expect = 1.7

Identities = 14/15 (93%)

Strand = Plus / Plus

Query: 143 gggcagaaccccact 157

|| ||||||||||||

Sbjct: 7 ggacagaaccccact 21

>zma-miR395l* MIMAT0015344

Length = 22

Score = 21.1 bits (22), Expect = 5.9

Identities = 11/11 (100%)

Strand = Plus / Plus

Query: 682 ttccttccaaa 692

|||||||||||

Sbjct: 2 ttccttccaaa 12

>tae-miR167b MIMAT0018214

Length = 21

Score = 21.1 bits (22), Expect = 5.9

Identities = 11/11 (100%)

Strand = Plus / Plus

Query: 367 gaagctgacag 377

|||||||||||

Sbjct: 2 gaagctgacag 12

>osa-miR1320-3p MIMAT0015286

Length = 21

Score = 21.1 bits (22), Expect = 5.9

Identities = 14/16 (87%)

Strand = Plus / Minus

Query: 205 tggaacgattgacttt 220

|||||||| ||| |||

Sbjct: 20 tggaacgaatgaattt 5

>osa-miR2091-5p MIMAT0010046

Length = 21

Score = 21.1 bits (22), Expect = 5.9

Identities = 11/11 (100%)

Strand = Plus / Plus

Query: 267 ccgaggaggag 277

|||||||||||

Sbjct: 10 ccgaggaggag 20

Score = 21.1 bits (22), Expect = 5.9

Identities = 14/16 (87%)

Strand = Plus / Minus

Query: 205 tggaacgattgacttt 220

|||||||| ||| |||

Sbjct: 20 tggaacgaatgaattt 5

Score = 21.1 bits (22), Expect = 5.9

Identities = 11/11 (100%)

Strand = Plus / Plus

Query: 267 ccgaggaggag 277

|||||||||||

Sbjct: 10 ccgaggaggag 20

>ptc-miR1449 MIMAT0006011

Length = 22

Score = 21.1 bits (22), Expect = 5.9

Identities = 11/11 (100%)

Strand = Plus / Plus

Query: 242 gcacgtaagat 252

|||||||||||

Sbjct: 7 gcacgtaagat 17

>aqc-miR408 MIMAT0012581

Length = 21

Score = 21.1 bits (22), Expect = 5.9

Identities = 11/11 (100%)

Strand = Plus / Minus

Query: 157 tgaggcagagc 167

|||||||||||

Sbjct: 12 tgaggcagagc 2

>gma-miR4393b MIMAT0018317

Length = 24

Score = 21.1 bits (22), Expect = 5.9

Identities = 14/16 (87%)

Strand = Plus / Plus

Query: 550 ttgaaaactgacagca 565

||||||| |||||||

Sbjct: 1 ttgaaaagggacagca 16

>aly-miR3445.1* MIMAT0017724

Length = 21

Score = 21.1 bits (22), Expect = 5.9

Identities = 15/16 (93%), Gaps = 1/16 (6%)

Strand = Plus / Minus

Query: 445 gtttgtgaaggtgatg 460

||||| ||||||||||

Sbjct: 20 gtttg-gaaggtgatg 6

>aly-miR390b* MIMAT0017536

Length = 21

Score = 21.1 bits (22), Expect = 5.9

Identities = 11/11 (100%)

Strand = Plus / Plus

Query: 220 tcctgagttcc 230

|||||||||||

Sbjct: 10 tcctgagttcc 20

>aly-miR3433* MIMAT0017685

Length = 21

Score = 21.1 bits (22), Expect = 5.9

Identities = 11/11 (100%)

Strand = Plus / Plus

Query: 123 gaactgtgatg 133

|||||||||||

Sbjct: 11 gaactgtgatg 21

>ppt-miR1059 MIMAT0005177

Length = 21

Score = 21.1 bits (22), Expect = 5.9

Identities = 11/11 (100%)

Strand = Plus / Minus

Query: 445 gtttgtgaagg 455

|||||||||||

Sbjct: 18 gtttgtgaagg 8

BLASTN 2.2.21 [Jun-14-2009]

Reference: Altschul, Stephen F., Thomas L. Madden, Alejandro A. Schaffer,

Jinghui Zhang, Zheng Zhang, Webb Miller, and David J. Lipman (1997),

"Gapped BLAST and PSI-BLAST: a new generation of protein database search

programs", Nucleic Acids Res. 25:3389-3402.

Query= gi|1754996|gb|U48690.1|TAU48690 Triticum aestivum calmodulin

TaCaM2-1 mRNA, complete cds

(1125 letters)

Database: matureREL16.txt

4142 sequences; 88,243 total letters

Searching..................................................done

Score E

Sequences producing significant alignments: (bits) Value

tae-miR1118 MIMAT0005353 43 2e-006

tae-miR1125 MIMAT0005360 39 2e-005

osa-miR1439 MIMAT0005993 32 0.004

osa-miR1436 MIMAT0005987 30 0.013

hvu-miR1436 MIMAT0018501 30 0.013

tae-miR1133 MIMAT0005368 27 0.16

tae-miR1132 MIMAT0005367 27 0.16

tae-miR1128 MIMAT0005363 25 0.54

osa-miR2920 MIMAT0014051 23 1.9

bdi-miR1127 MIMAT0012190 23 1.9

mtr-miR2611 MIMAT0013320 23 1.9

aly-miR829 MIMAT0017612 23 1.9

zma-miR393b* MIMAT0015334 21 6.6

osa-miR812j MIMAT0007850 21 6.6

osa-miR812h MIMAT0007848 21 6.6

osa-miR812i MIMAT0007849 21 6.6

osa-miR1320 MIMAT0009137 21 6.6

osa-miR813 MIMAT0004054 21 6.6

osa-miR812f MIMAT0007821 21 6.6

osa-miR812g MIMAT0007847 21 6.6

osa-miR812j MIMAT0007850 21 6.6

osa-miR812h MIMAT0007848 21 6.6

osa-miR812i MIMAT0007849 21 6.6

osa-miR1320 MIMAT0009137 21 6.6

osa-miR813 MIMAT0004054 21 6.6

osa-miR812f MIMAT0007821 21 6.6

osa-miR812g MIMAT0007847 21 6.6

aqc-miR408 MIMAT0012581 21 6.6

mtr-miR2588a MIMAT0013258 21 6.6

mtr-miR2588b MIMAT0013259 21 6.6

aly-miR846* MIMAT0017635 21 6.6

aly-miR4249 MIMAT0017940 21 6.6

aly-miR3445.1 MIMAT0017725 21 6.6

aly-miR4233 MIMAT0017922 21 6.6

aly-miR160c* MIMAT0017440 21 6.6

smo-miR1084 MIMAT0005235 21 6.6

ppt-miR2079 MIMAT0010016 21 6.6

ppt-miR1216 MIMAT0003905 21 6.6

>tae-miR1118 MIMAT0005353

Length = 23

Score = 42.8 bits (46), Expect = 2e-006

Identities = 23/23 (100%)

Strand = Plus / Plus

Query: 938 cactacattatggaatggaggga 960

|||||||||||||||||||||||

Sbjct: 1 cactacattatggaatggaggga 23

Score = 28.3 bits (30), Expect = 0.045

Identities = 20/23 (86%)

Strand = Plus / Minus

Query: 726 tccctccgttccaaaatatagtg 748

||||||| ||||| ||| |||||

Sbjct: 23 tccctccattccataatgtagtg 1

>tae-miR1125 MIMAT0005360

Length = 24

Score = 39.2 bits (42), Expect = 2e-005

Identities = 23/24 (95%)

Strand = Plus / Plus

Query: 788 aaccaatgagaccaactgcggcgg 811

|||||| |||||||||||||||||

Sbjct: 1 aaccaacgagaccaactgcggcgg 24

Score = 21.1 bits (22), Expect = 6.6

Identities = 19/24 (79%)

Strand = Plus / Minus

Query: 875 ccgccgcaatcggtcttggtagtt 898

|||||||| | ||||| | | |||

Sbjct: 24 ccgccgcagttggtctcgttggtt 1

>osa-miR1439 MIMAT0005993

Length = 21

Score = 31.9 bits (34), Expect = 0.004

Identities = 19/20 (95%)

Strand = Plus / Minus

Query: 722 atactccctccgttccaaaa 741

|||||| |||||||||||||

Sbjct: 20 atactcactccgttccaaaa 1

Score = 31.9 bits (34), Expect = 0.004

Identities = 19/20 (95%)

Strand = Plus / Minus

Query: 722 atactccctccgttccaaaa 741

|||||| |||||||||||||

Sbjct: 20 atactcactccgttccaaaa 1

>osa-miR1436 MIMAT0005987

Length = 21

Score = 30.1 bits (32), Expect = 0.013

Identities = 19/21 (90%)

Strand = Plus / Plus

Query: 942 acattatggaatggagggagt 962

||||||||| | |||||||||

Sbjct: 1 acattatgggacggagggagt 21

Score = 26.5 bits (28), Expect = 0.16

Identities = 17/19 (89%)

Strand = Plus / Minus

Query: 724 actccctccgttccaaaat 742

||||||||||| ||| |||

Sbjct: 21 actccctccgtcccataat 3

Score = 30.1 bits (32), Expect = 0.013

Identities = 19/21 (90%)

Strand = Plus / Plus

Query: 942 acattatggaatggagggagt 962

||||||||| | |||||||||

Sbjct: 1 acattatgggacggagggagt 21

Score = 26.5 bits (28), Expect = 0.16

Identities = 17/19 (89%)

Strand = Plus / Minus

Query: 724 actccctccgttccaaaat 742

||||||||||| ||| |||

Sbjct: 21 actccctccgtcccataat 3

>hvu-miR1436 MIMAT0018501

Length = 21

Score = 30.1 bits (32), Expect = 0.013

Identities = 19/21 (90%)

Strand = Plus / Plus

Query: 942 acattatggaatggagggagt 962

||||||||| | |||||||||

Sbjct: 1 acattatgggacggagggagt 21

Score = 26.5 bits (28), Expect = 0.16

Identities = 17/19 (89%)

Strand = Plus / Minus

Query: 724 actccctccgttccaaaat 742

||||||||||| ||| |||

Sbjct: 21 actccctccgtcccataat 3

>tae-miR1133 MIMAT0005368

Length = 22

Score = 26.5 bits (28), Expect = 0.16

Identities = 17/19 (89%)

Strand = Plus / Plus

Query: 722 atactccctccgttccaaa 740

||||||||||||| | |||

Sbjct: 4 atactccctccgtccgaaa 22

Score = 21.1 bits (22), Expect = 6.6

Identities = 11/11 (100%)

Strand = Plus / Minus

Query: 954 ggagggagtat 964

|||||||||||

Sbjct: 14 ggagggagtat 4

>tae-miR1132 MIMAT0005367

Length = 19

Score = 26.5 bits (28), Expect = 0.16

Identities = 17/19 (89%)

Strand = Plus / Plus

Query: 943 cattatggaatggagggag 961

|||||||||| ||| ||||

Sbjct: 1 cattatggaacggaaggag 19

Score = 24.7 bits (26), Expect = 0.54

Identities = 16/18 (88%)

Strand = Plus / Minus

Query: 725 ctccctccgttccaaaat 742

|||| ||||||||| |||

Sbjct: 19 ctccttccgttccataat 2

>tae-miR1128 MIMAT0005363

Length = 21

Score = 24.7 bits (26), Expect = 0.54

Identities = 16/18 (88%)

Strand = Plus / Plus

Query: 723 tactccctccgttccaaa 740

|||||||||||| | |||

Sbjct: 4 tactccctccgtccgaaa 21

>osa-miR2920 MIMAT0014051

Length = 23

Score = 22.9 bits (24), Expect = 1.9

Identities = 12/12 (100%)

Strand = Plus / Plus

Query: 698 aacaatataaca 709

||||||||||||

Sbjct: 5 aacaatataaca 16

Score = 22.9 bits (24), Expect = 1.9

Identities = 12/12 (100%)

Strand = Plus / Plus

Query: 698 aacaatataaca 709

||||||||||||

Sbjct: 5 aacaatataaca 16

>bdi-miR1127 MIMAT0012190

Length = 21

Score = 22.9 bits (24), Expect = 1.9

Identities = 12/12 (100%)

Strand = Plus / Plus

Query: 723 tactccctccgt 734

||||||||||||

Sbjct: 4 tactccctccgt 15

>mtr-miR2611 MIMAT0013320

Length = 21

Score = 22.9 bits (24), Expect = 1.9

Identities = 17/20 (85%)

Strand = Plus / Plus

Query: 1101 tatatgtcagtgttccatga 1120

||| |||||||||| ||||

Sbjct: 1 tatttgtcagtgtttgatga 20

>aly-miR829 MIMAT0017612

Length = 21

Score = 22.9 bits (24), Expect = 1.9

Identities = 14/15 (93%)

Strand = Plus / Minus

Query: 986 ccttgaagattttat 1000

|||||||||||| ||

Sbjct: 18 ccttgaagatttgat 4

>zma-miR393b* MIMAT0015334

Length = 22

Score = 21.1 bits (22), Expect = 6.6

Identities = 14/16 (87%)

Strand = Plus / Plus

Query: 141 tgagatccgtttggag 156

|| ||||| |||||||

Sbjct: 6 tgcgatccttttggag 21

>osa-miR812j MIMAT0007850

Length = 24

Score = 21.1 bits (22), Expect = 6.6

Identities = 14/16 (87%)

Strand = Plus / Minus

Query: 766 gtccaactttgaccat 781

|||||||||| | |||

Sbjct: 23 gtccaactttaatcat 8

>osa-miR812h MIMAT0007848

Length = 24

Score = 21.1 bits (22), Expect = 6.6

Identities = 14/16 (87%)

Strand = Plus / Minus

Query: 766 gtccaactttgaccat 781

|||||||||| | |||

Sbjct: 23 gtccaactttaatcat 8

>osa-miR812i MIMAT0007849

Length = 24

Score = 21.1 bits (22), Expect = 6.6

Identities = 14/16 (87%)

Strand = Plus / Minus

Query: 766 gtccaactttgaccat 781

|||||||||| | |||

Sbjct: 23 gtccaactttaatcat 8

>osa-miR1320 MIMAT0009137

Length = 21

Score = 21.1 bits (22), Expect = 6.6

Identities = 11/11 (100%)

Strand = Plus / Minus

Query: 728 cctccgttcca 738

|||||||||||

Sbjct: 11 cctccgttcca 1

>osa-miR813 MIMAT0004054

Length = 22

Score = 21.1 bits (22), Expect = 6.6

Identities = 11/11 (100%)

Strand = Plus / Plus

Query: 945 ttatggaatgg 955

|||||||||||

Sbjct: 4 ttatggaatgg 14

>osa-miR812f MIMAT0007821

Length = 24

Score = 21.1 bits (22), Expect = 6.6

Identities = 14/16 (87%)

Strand = Plus / Minus

Query: 766 gtccaactttgaccat 781

|||||||||| | |||

Sbjct: 20 gtccaactttaatcat 5

>osa-miR812g MIMAT0007847

Length = 24

Score = 21.1 bits (22), Expect = 6.6

Identities = 14/16 (87%)

Strand = Plus / Minus

Query: 766 gtccaactttgaccat 781

|||||||||| | |||

Sbjct: 23 gtccaactttaatcat 8

Score = 21.1 bits (22), Expect = 6.6

Identities = 14/16 (87%)

Strand = Plus / Minus

Query: 766 gtccaactttgaccat 781

|||||||||| | |||

Sbjct: 23 gtccaactttaatcat 8

Score = 21.1 bits (22), Expect = 6.6

Identities = 14/16 (87%)

Strand = Plus / Minus

Query: 766 gtccaactttgaccat 781

|||||||||| | |||

Sbjct: 23 gtccaactttaatcat 8

Score = 21.1 bits (22), Expect = 6.6

Identities = 14/16 (87%)

Strand = Plus / Minus

Query: 766 gtccaactttgaccat 781

|||||||||| | |||

Sbjct: 23 gtccaactttaatcat 8

Score = 21.1 bits (22), Expect = 6.6

Identities = 11/11 (100%)

Strand = Plus / Minus

Query: 728 cctccgttcca 738

|||||||||||

Sbjct: 11 cctccgttcca 1

Score = 21.1 bits (22), Expect = 6.6

Identities = 11/11 (100%)

Strand = Plus / Plus

Query: 945 ttatggaatgg 955

|||||||||||

Sbjct: 4 ttatggaatgg 14

Score = 21.1 bits (22), Expect = 6.6

Identities = 14/16 (87%)

Strand = Plus / Minus

Query: 766 gtccaactttgaccat 781

|||||||||| | |||

Sbjct: 20 gtccaactttaatcat 5

Score = 21.1 bits (22), Expect = 6.6

Identities = 14/16 (87%)

Strand = Plus / Minus

Query: 766 gtccaactttgaccat 781

|||||||||| | |||

Sbjct: 23 gtccaactttaatcat 8

>aqc-miR408 MIMAT0012581

Length = 21

Score = 21.1 bits (22), Expect = 6.6

Identities = 11/11 (100%)

Strand = Plus / Minus

Query: 288 tgaggcagagc 298

|||||||||||

Sbjct: 12 tgaggcagagc 2

>mtr-miR2588a MIMAT0013258

Length = 21

Score = 21.1 bits (22), Expect = 6.6

Identities = 11/11 (100%)

Strand = Plus / Plus

Query: 705 taacactgtgc 715

|||||||||||

Sbjct: 1 taacactgtgc 11

>mtr-miR2588b MIMAT0013259

Length = 21

Score = 21.1 bits (22), Expect = 6.6

Identities = 11/11 (100%)

Strand = Plus / Plus

Query: 705 taacactgtgc 715

|||||||||||

Sbjct: 1 taacactgtgc 11

>aly-miR846* MIMAT0017635

Length = 21

Score = 21.1 bits (22), Expect = 6.6

Identities = 14/16 (87%)

Strand = Plus / Minus

Query: 342 aattgatttccctgaa 357

|||||| ||||||||

Sbjct: 16 aattgaagtccctgaa 1

>aly-miR4249 MIMAT0017940

Length = 21

Score = 21.1 bits (22), Expect = 6.6

Identities = 11/11 (100%)

Strand = Plus / Plus

Query: 495 tgagaagttga 505

|||||||||||

Sbjct: 7 tgagaagttga 17

>aly-miR3445.1 MIMAT0017725

Length = 21

Score = 21.1 bits (22), Expect = 6.6

Identities = 11/11 (100%)

Strand = Plus / Plus

Query: 976 tgattgtttgc 986

|||||||||||

Sbjct: 10 tgattgtttgc 20

>aly-miR4233 MIMAT0017922

Length = 21

Score = 21.1 bits (22), Expect = 6.6

Identities = 13/14 (92%)

Strand = Plus / Minus

Query: 540 tgatgttgatggtg 553

||||||||||| ||

Sbjct: 16 tgatgttgatgatg 3

>aly-miR160c* MIMAT0017440

Length = 21

Score = 21.1 bits (22), Expect = 6.6

Identities = 14/16 (87%)

Strand = Plus / Minus

Query: 175 atgcttgactgcttgt 190

||||||| || |||||

Sbjct: 20 atgcttggctccttgt 5

>smo-miR1084 MIMAT0005235

Length = 22

Score = 21.1 bits (22), Expect = 6.6

Identities = 11/11 (100%)

Strand = Plus / Minus

Query: 611 taccacctgag 621

|||||||||||

Sbjct: 14 taccacctgag 4

>ppt-miR2079 MIMAT0010016

Length = 21

Score = 21.1 bits (22), Expect = 6.6

Identities = 11/11 (100%)

Strand = Plus / Plus

Query: 540 tgatgttgatg 550

|||||||||||

Sbjct: 6 tgatgttgatg 16

>ppt-miR1216 MIMAT0003905

Length = 21

Score = 21.1 bits (22), Expect = 6.6

Identities = 11/11 (100%)

Strand = Plus / Plus

Query: 546 tgatggtgatg 556

|||||||||||

Sbjct: 1 tgatggtgatg 11

BLASTN 2.2.21 [Jun-14-2009]

Reference: Altschul, Stephen F., Thomas L. Madden, Alejandro A. Schaffer,

Jinghui Zhang, Zheng Zhang, Webb Miller, and David J. Lipman (1997),

"Gapped BLAST and PSI-BLAST: a new generation of protein database search

programs", Nucleic Acids Res. 25:3389-3402.

Query= gi|1754998|gb|U48691.1|TAU48691 Triticum aestivum calmodulin

TaCaM2-2 mRNA, complete cds

(1023 letters)

Database: matureREL16.txt

4142 sequences; 88,243 total letters

Searching..................................................done

Score E

Sequences producing significant alignments: (bits) Value

tae-miR1118 MIMAT0005353 43 2e-006

tae-miR1125 MIMAT0005360 39 3e-005

osa-miR1439 MIMAT0005993 32 0.004

osa-miR1436 MIMAT0005987 30 0.015

hvu-miR1436 MIMAT0018501 30 0.015

tae-miR1133 MIMAT0005368 27 0.18

tae-miR1132 MIMAT0005367 27 0.18

tae-miR1128 MIMAT0005363 25 0.64

osa-miR2920 MIMAT0014051 23 2.2

bdi-miR1127 MIMAT0012190 23 2.2

mtr-miR2611 MIMAT0013320 23 2.2

aly-miR829 MIMAT0017612 23 2.2

osa-miR812j MIMAT0007850 21 7.9

osa-miR812h MIMAT0007848 21 7.9

osa-miR812i MIMAT0007849 21 7.9

osa-miR1320 MIMAT0009137 21 7.9

osa-miR813 MIMAT0004054 21 7.9

osa-miR812f MIMAT0007821 21 7.9

osa-miR812g MIMAT0007847 21 7.9

osa-miR812j MIMAT0007850 21 7.9

osa-miR812h MIMAT0007848 21 7.9

osa-miR812i MIMAT0007849 21 7.9

osa-miR1320 MIMAT0009137 21 7.9

osa-miR813 MIMAT0004054 21 7.9

osa-miR812f MIMAT0007821 21 7.9

osa-miR812g MIMAT0007847 21 7.9

aqc-miR408 MIMAT0012581 21 7.9

mtr-miR2588a MIMAT0013258 21 7.9

mtr-miR2588b MIMAT0013259 21 7.9

aly-miR846* MIMAT0017635 21 7.9

aly-miR4249 MIMAT0017940 21 7.9

aly-miR3445.1 MIMAT0017725 21 7.9

aly-miR4233 MIMAT0017922 21 7.9

ppt-miR2079 MIMAT0010016 21 7.9

ppt-miR1216 MIMAT0003905 21 7.9

>tae-miR1118 MIMAT0005353

Length = 23

Score = 42.8 bits (46), Expect = 2e-006

Identities = 23/23 (100%)

Strand = Plus / Plus

Query: 841 cactacattatggaatggaggga 863

|||||||||||||||||||||||

Sbjct: 1 cactacattatggaatggaggga 23

Score = 28.3 bits (30), Expect = 0.053

Identities = 20/23 (86%)

Strand = Plus / Minus

Query: 629 tccctccgttccaaaatatagtg 651

||||||| ||||| ||| |||||

Sbjct: 23 tccctccattccataatgtagtg 1

>tae-miR1125 MIMAT0005360

Length = 24

Score = 39.2 bits (42), Expect = 3e-005

Identities = 23/24 (95%)

Strand = Plus / Plus

Query: 691 aaccaatgagaccaactgcggcgg 714

|||||| |||||||||||||||||

Sbjct: 1 aaccaacgagaccaactgcggcgg 24

Score = 21.1 bits (22), Expect = 7.9

Identities = 19/24 (79%)

Strand = Plus / Minus

Query: 778 ccgccgcaatcggtcttggtagtt 801

|||||||| | ||||| | | |||

Sbjct: 24 ccgccgcagttggtctcgttggtt 1

>osa-miR1439 MIMAT0005993

Length = 21

Score = 31.9 bits (34), Expect = 0.004

Identities = 19/20 (95%)

Strand = Plus / Minus

Query: 625 atactccctccgttccaaaa 644

|||||| |||||||||||||

Sbjct: 20 atactcactccgttccaaaa 1

Score = 31.9 bits (34), Expect = 0.004

Identities = 19/20 (95%)

Strand = Plus / Minus

Query: 625 atactccctccgttccaaaa 644

|||||| |||||||||||||

Sbjct: 20 atactcactccgttccaaaa 1

>osa-miR1436 MIMAT0005987

Length = 21

Score = 30.1 bits (32), Expect = 0.015

Identities = 19/21 (90%)

Strand = Plus / Plus

Query: 845 acattatggaatggagggagt 865

||||||||| | |||||||||

Sbjct: 1 acattatgggacggagggagt 21

Score = 26.5 bits (28), Expect = 0.18

Identities = 17/19 (89%)

Strand = Plus / Minus

Query: 627 actccctccgttccaaaat 645

||||||||||| ||| |||

Sbjct: 21 actccctccgtcccataat 3

Score = 30.1 bits (32), Expect = 0.015

Identities = 19/21 (90%)

Strand = Plus / Plus

Query: 845 acattatggaatggagggagt 865

||||||||| | |||||||||

Sbjct: 1 acattatgggacggagggagt 21

Score = 26.5 bits (28), Expect = 0.18

Identities = 17/19 (89%)

Strand = Plus / Minus

Query: 627 actccctccgttccaaaat 645

||||||||||| ||| |||

Sbjct: 21 actccctccgtcccataat 3

>hvu-miR1436 MIMAT0018501

Length = 21

Score = 30.1 bits (32), Expect = 0.015

Identities = 19/21 (90%)

Strand = Plus / Plus

Query: 845 acattatggaatggagggagt 865

||||||||| | |||||||||

Sbjct: 1 acattatgggacggagggagt 21

Score = 26.5 bits (28), Expect = 0.18

Identities = 17/19 (89%)

Strand = Plus / Minus

Query: 627 actccctccgttccaaaat 645

||||||||||| ||| |||

Sbjct: 21 actccctccgtcccataat 3

>tae-miR1133 MIMAT0005368

Length = 22

Score = 26.5 bits (28), Expect = 0.18

Identities = 17/19 (89%)

Strand = Plus / Plus

Query: 625 atactccctccgttccaaa 643

||||||||||||| | |||

Sbjct: 4 atactccctccgtccgaaa 22

Score = 21.1 bits (22), Expect = 7.9

Identities = 11/11 (100%)

Strand = Plus / Minus

Query: 857 ggagggagtat 867

|||||||||||

Sbjct: 14 ggagggagtat 4

>tae-miR1132 MIMAT0005367

Length = 19

Score = 26.5 bits (28), Expect = 0.18

Identities = 17/19 (89%)

Strand = Plus / Plus

Query: 846 cattatggaatggagggag 864

|||||||||| ||| ||||

Sbjct: 1 cattatggaacggaaggag 19

Score = 24.7 bits (26), Expect = 0.64

Identities = 16/18 (88%)

Strand = Plus / Minus

Query: 628 ctccctccgttccaaaat 645

|||| ||||||||| |||

Sbjct: 19 ctccttccgttccataat 2

>tae-miR1128 MIMAT0005363

Length = 21

Score = 24.7 bits (26), Expect = 0.64

Identities = 16/18 (88%)

Strand = Plus / Plus

Query: 626 tactccctccgttccaaa 643

|||||||||||| | |||

Sbjct: 4 tactccctccgtccgaaa 21

>osa-miR2920 MIMAT0014051

Length = 23

Score = 22.9 bits (24), Expect = 2.2

Identities = 12/12 (100%)

Strand = Plus / Plus

Query: 601 aacaatataaca 612

||||||||||||

Sbjct: 5 aacaatataaca 16

Score = 22.9 bits (24), Expect = 2.2

Identities = 12/12 (100%)

Strand = Plus / Plus

Query: 601 aacaatataaca 612

||||||||||||

Sbjct: 5 aacaatataaca 16

>bdi-miR1127 MIMAT0012190

Length = 21

Score = 22.9 bits (24), Expect = 2.2

Identities = 12/12 (100%)

Strand = Plus / Plus

Query: 626 tactccctccgt 637

||||||||||||

Sbjct: 4 tactccctccgt 15

>mtr-miR2611 MIMAT0013320

Length = 21

Score = 22.9 bits (24), Expect = 2.2

Identities = 17/20 (85%)

Strand = Plus / Plus

Query: 1004 tatatgtcagtgttccatga 1023

||| |||||||||| ||||

Sbjct: 1 tatttgtcagtgtttgatga 20

>aly-miR829 MIMAT0017612

Length = 21

Score = 22.9 bits (24), Expect = 2.2

Identities = 14/15 (93%)

Strand = Plus / Minus

Query: 889 ccttgaagattttat 903

|||||||||||| ||

Sbjct: 18 ccttgaagatttgat 4

>osa-miR812j MIMAT0007850

Length = 24

Score = 21.1 bits (22), Expect = 7.9

Identities = 14/16 (87%)

Strand = Plus / Minus

Query: 669 gtccaactttgaccat 684

|||||||||| | |||

Sbjct: 23 gtccaactttaatcat 8

>osa-miR812h MIMAT0007848

Length = 24

Score = 21.1 bits (22), Expect = 7.9

Identities = 14/16 (87%)

Strand = Plus / Minus

Query: 669 gtccaactttgaccat 684

|||||||||| | |||

Sbjct: 23 gtccaactttaatcat 8

>osa-miR812i MIMAT0007849

Length = 24

Score = 21.1 bits (22), Expect = 7.9

Identities = 14/16 (87%)

Strand = Plus / Minus

Query: 669 gtccaactttgaccat 684

|||||||||| | |||

Sbjct: 23 gtccaactttaatcat 8

>osa-miR1320 MIMAT0009137

Length = 21

Score = 21.1 bits (22), Expect = 7.9

Identities = 11/11 (100%)

Strand = Plus / Minus

Query: 631 cctccgttcca 641

|||||||||||

Sbjct: 11 cctccgttcca 1

>osa-miR813 MIMAT0004054

Length = 22

Score = 21.1 bits (22), Expect = 7.9

Identities = 11/11 (100%)

Strand = Plus / Plus

Query: 848 ttatggaatgg 858

|||||||||||

Sbjct: 4 ttatggaatgg 14

>osa-miR812f MIMAT0007821

Length = 24

Score = 21.1 bits (22), Expect = 7.9

Identities = 14/16 (87%)

Strand = Plus / Minus

Query: 669 gtccaactttgaccat 684

|||||||||| | |||

Sbjct: 20 gtccaactttaatcat 5

>osa-miR812g MIMAT0007847

Length = 24

Score = 21.1 bits (22), Expect = 7.9

Identities = 14/16 (87%)

Strand = Plus / Minus

Query: 669 gtccaactttgaccat 684

|||||||||| | |||

Sbjct: 23 gtccaactttaatcat 8

Score = 21.1 bits (22), Expect = 7.9

Identities = 14/16 (87%)

Strand = Plus / Minus

Query: 669 gtccaactttgaccat 684

|||||||||| | |||

Sbjct: 23 gtccaactttaatcat 8

Score = 21.1 bits (22), Expect = 7.9

Identities = 14/16 (87%)

Strand = Plus / Minus

Query: 669 gtccaactttgaccat 684

|||||||||| | |||

Sbjct: 23 gtccaactttaatcat 8

Score = 21.1 bits (22), Expect = 7.9

Identities = 14/16 (87%)

Strand = Plus / Minus

Query: 669 gtccaactttgaccat 684

|||||||||| | |||

Sbjct: 23 gtccaactttaatcat 8

Score = 21.1 bits (22), Expect = 7.9

Identities = 11/11 (100%)

Strand = Plus / Minus

Query: 631 cctccgttcca 641

|||||||||||

Sbjct: 11 cctccgttcca 1

Score = 21.1 bits (22), Expect = 7.9

Identities = 11/11 (100%)

Strand = Plus / Plus

Query: 848 ttatggaatgg 858

|||||||||||

Sbjct: 4 ttatggaatgg 14

Score = 21.1 bits (22), Expect = 7.9

Identities = 14/16 (87%)

Strand = Plus / Minus

Query: 669 gtccaactttgaccat 684

|||||||||| | |||

Sbjct: 20 gtccaactttaatcat 5

Score = 21.1 bits (22), Expect = 7.9

Identities = 14/16 (87%)

Strand = Plus / Minus

Query: 669 gtccaactttgaccat 684

|||||||||| | |||

Sbjct: 23 gtccaactttaatcat 8

>aqc-miR408 MIMAT0012581

Length = 21

Score = 21.1 bits (22), Expect = 7.9

Identities = 11/11 (100%)

Strand = Plus / Minus

Query: 191 tgaggcagagc 201

|||||||||||

Sbjct: 12 tgaggcagagc 2

>mtr-miR2588a MIMAT0013258

Length = 21

Score = 21.1 bits (22), Expect = 7.9

Identities = 11/11 (100%)

Strand = Plus / Plus

Query: 608 taacactgtgc 618

|||||||||||

Sbjct: 1 taacactgtgc 11

>mtr-miR2588b MIMAT0013259

Length = 21

Score = 21.1 bits (22), Expect = 7.9

Identities = 11/11 (100%)

Strand = Plus / Plus

Query: 608 taacactgtgc 618

|||||||||||

Sbjct: 1 taacactgtgc 11

>aly-miR846* MIMAT0017635

Length = 21

Score = 21.1 bits (22), Expect = 7.9

Identities = 14/16 (87%)

Strand = Plus / Minus

Query: 245 aattgatttccctgaa 260

|||||| ||||||||

Sbjct: 16 aattgaagtccctgaa 1

>aly-miR4249 MIMAT0017940

Length = 21

Score = 21.1 bits (22), Expect = 7.9

Identities = 11/11 (100%)

Strand = Plus / Plus

Query: 398 tgagaagttga 408

|||||||||||

Sbjct: 7 tgagaagttga 17

>aly-miR3445.1 MIMAT0017725

Length = 21

Score = 21.1 bits (22), Expect = 7.9

Identities = 11/11 (100%)

Strand = Plus / Plus

Query: 879 tgattgtttgc 889

|||||||||||

Sbjct: 10 tgattgtttgc 20

>aly-miR4233 MIMAT0017922

Length = 21

Score = 21.1 bits (22), Expect = 7.9

Identities = 13/14 (92%)

Strand = Plus / Minus

Query: 443 tgatgttgatggtg 456

||||||||||| ||

Sbjct: 16 tgatgttgatgatg 3

>ppt-miR2079 MIMAT0010016

Length = 21

Score = 21.1 bits (22), Expect = 7.9

Identities = 11/11 (100%)

Strand = Plus / Plus

Query: 443 tgatgttgatg 453

|||||||||||

Sbjct: 6 tgatgttgatg 16

>ppt-miR1216 MIMAT0003905

Length = 21

Score = 21.1 bits (22), Expect = 7.9

Identities = 11/11 (100%)

Strand = Plus / Plus

Query: 449 tgatggtgatg 459

|||||||||||

Sbjct: 1 tgatggtgatg 11

BLASTN 2.2.21 [Jun-14-2009]

Reference: Altschul, Stephen F., Thomas L. Madden, Alejandro A. Schaffer,

Jinghui Zhang, Zheng Zhang, Webb Miller, and David J. Lipman (1997),

"Gapped BLAST and PSI-BLAST: a new generation of protein database search

programs", Nucleic Acids Res. 25:3389-3402.

Query= gi|1755000|gb|U48692.1|TAU48692 Triticum aestivum calmodulin

TaCaM2-3 mRNA, complete cds

(909 letters)

Database: matureREL16.txt

4142 sequences; 88,243 total letters

Searching..................................................done

Score E

Sequences producing significant alignments: (bits) Value

osa-miR2920 MIMAT0014051 23 2.0

mtr-miR2611 MIMAT0013320 23 2.0

aly-miR829 MIMAT0017612 23 2.0

osa-miR2867 MIMAT0013814 21 7.0

vvi-miR828b MIMAT0006578 21 7.0

aqc-miR408 MIMAT0012581 21 7.0

mtr-miR2588a MIMAT0013258 21 7.0

mtr-miR2588b MIMAT0013259 21 7.0

ahy-miR3513-5p MIMAT0016337 21 7.0

aly-miR846* MIMAT0017635 21 7.0

aly-miR4249 MIMAT0017940 21 7.0

aly-miR3445.1 MIMAT0017725 21 7.0

aly-miR4233 MIMAT0017922 21 7.0

smo-miR1084 MIMAT0005235 21 7.0

ppt-miR2079 MIMAT0010016 21 7.0

ppt-miR1216 MIMAT0003905 21 7.0

>osa-miR2920 MIMAT0014051

Length = 23

Score = 22.9 bits (24), Expect = 2.0

Identities = 12/12 (100%)

Strand = Plus / Plus

Query: 613 aacaatataaca 624

||||||||||||

Sbjct: 5 aacaatataaca 16

Score = 22.9 bits (24), Expect = 2.0

Identities = 15/17 (88%)

Strand = Plus / Minus

Query: 809 ttgaagtgttatactgt 825

||||| ||||||| |||

Sbjct: 22 ttgaaatgttatattgt 6

Score = 22.9 bits (24), Expect = 2.0

Identities = 12/12 (100%)

Strand = Plus / Plus

Query: 613 aacaatataaca 624

||||||||||||

Sbjct: 5 aacaatataaca 16

Score = 22.9 bits (24), Expect = 2.0

Identities = 15/17 (88%)

Strand = Plus / Minus

Query: 809 ttgaagtgttatactgt 825

||||| ||||||| |||

Sbjct: 22 ttgaaatgttatattgt 6

>mtr-miR2611 MIMAT0013320

Length = 21

Score = 22.9 bits (24), Expect = 2.0

Identities = 17/20 (85%)

Strand = Plus / Plus

Query: 775 tatatgtcagtgttccatga 794

||| |||||||||| ||||

Sbjct: 1 tatttgtcagtgtttgatga 20

>aly-miR829 MIMAT0017612

Length = 21

Score = 22.9 bits (24), Expect = 2.0

Identities = 14/15 (93%)

Strand = Plus / Minus

Query: 662 ccttgaagattttat 676

|||||||||||| ||

Sbjct: 18 ccttgaagatttgat 4

>osa-miR2867 MIMAT0013814

Length = 22

Score = 21.1 bits (22), Expect = 7.0

Identities = 11/11 (100%)

Strand = Plus / Minus

Query: 585 tgtgggatggc 595

|||||||||||

Sbjct: 14 tgtgggatggc 4

Score = 21.1 bits (22), Expect = 7.0

Identities = 11/11 (100%)

Strand = Plus / Minus

Query: 585 tgtgggatggc 595

|||||||||||

Sbjct: 14 tgtgggatggc 4

>vvi-miR828b MIMAT0006578

Length = 22

Score = 21.1 bits (22), Expect = 7.0

Identities = 11/11 (100%)

Strand = Plus / Minus

Query: 2 gaacactcatt 12

|||||||||||

Sbjct: 20 gaacactcatt 10

>aqc-miR408 MIMAT0012581

Length = 21

Score = 21.1 bits (22), Expect = 7.0

Identities = 11/11 (100%)

Strand = Plus / Minus

Query: 203 tgaggcagagc 213

|||||||||||

Sbjct: 12 tgaggcagagc 2

>mtr-miR2588a MIMAT0013258

Length = 21

Score = 21.1 bits (22), Expect = 7.0

Identities = 11/11 (100%)

Strand = Plus / Plus

Query: 620 taacactgtgc 630

|||||||||||

Sbjct: 1 taacactgtgc 11

>mtr-miR2588b MIMAT0013259

Length = 21

Score = 21.1 bits (22), Expect = 7.0

Identities = 11/11 (100%)

Strand = Plus / Plus

Query: 620 taacactgtgc 630

|||||||||||

Sbjct: 1 taacactgtgc 11

>ahy-miR3513-5p MIMAT0016337

Length = 21

Score = 21.1 bits (22), Expect = 7.0

Identities = 13/14 (92%)

Strand = Plus / Plus

Query: 693 tttctgattttgtc 706

||||||| ||||||

Sbjct: 5 tttctgagtttgtc 18

>aly-miR846* MIMAT0017635

Length = 21

Score = 21.1 bits (22), Expect = 7.0

Identities = 14/16 (87%)

Strand = Plus / Minus

Query: 257 aattgatttccctgaa 272

|||||| ||||||||

Sbjct: 16 aattgaagtccctgaa 1

>aly-miR4249 MIMAT0017940

Length = 21

Score = 21.1 bits (22), Expect = 7.0

Identities = 11/11 (100%)

Strand = Plus / Plus

Query: 410 tgagaagttga 420

|||||||||||

Sbjct: 7 tgagaagttga 17

>aly-miR3445.1 MIMAT0017725

Length = 21

Score = 21.1 bits (22), Expect = 7.0

Identities = 11/11 (100%)

Strand = Plus / Plus

Query: 652 tgattgtttgc 662

|||||||||||

Sbjct: 10 tgattgtttgc 20

>aly-miR4233 MIMAT0017922

Length = 21

Score = 21.1 bits (22), Expect = 7.0

Identities = 13/14 (92%)

Strand = Plus / Minus

Query: 455 tgatgttgatggtg 468

||||||||||| ||

Sbjct: 16 tgatgttgatgatg 3

>smo-miR1084 MIMAT0005235

Length = 22

Score = 21.1 bits (22), Expect = 7.0

Identities = 11/11 (100%)

Strand = Plus / Minus

Query: 526 taccacctgag 536

|||||||||||

Sbjct: 14 taccacctgag 4

>ppt-miR2079 MIMAT0010016

Length = 21

Score = 21.1 bits (22), Expect = 7.0

Identities = 11/11 (100%)

Strand = Plus / Plus

Query: 455 tgatgttgatg 465

|||||||||||

Sbjct: 6 tgatgttgatg 16

>ppt-miR1216 MIMAT0003905

Length = 21

Score = 21.1 bits (22), Expect = 7.0

Identities = 11/11 (100%)

Strand = Plus / Plus

Query: 461 tgatggtgatg 471

|||||||||||

Sbjct: 1 tgatggtgatg 11

BLASTN 2.2.21 [Jun-14-2009]

Reference: Altschul, Stephen F., Thomas L. Madden, Alejandro A. Schaffer,

Jinghui Zhang, Zheng Zhang, Webb Miller, and David J. Lipman (1997),

"Gapped BLAST and PSI-BLAST: a new generation of protein database search

programs", Nucleic Acids Res. 25:3389-3402.

Query= gi|1755002|gb|U48693.1|TAU48693 Triticum aestivum calmodulin

TaCaM3-1 mRNA, complete cds

(1529 letters)

Database: matureREL16.txt

4142 sequences; 88,243 total letters

Searching..................................................done

Score E

Sequences producing significant alignments: (bits) Value

mtr-miR2630n MIMAT0013386 25 0.74

mtr-miR2630y MIMAT0013375 25 0.74

mtr-miR2630a MIMAT0013370 25 0.74

mtr-miR2630k MIMAT0013383 25 0.74

mtr-miR2630p MIMAT0013388 25 0.74

mtr-miR2630x MIMAT0013374 25 0.74

mtr-miR2630h MIMAT0013380 25 0.74

mtr-miR2630m MIMAT0013385 25 0.74

mtr-miR2630w MIMAT0013373 25 0.74

mtr-miR2630f MIMAT0013378 25 0.74

mtr-miR2630s MIMAT0013391 25 0.74

mtr-miR2630j MIMAT0013382 25 0.74

mtr-miR2630o MIMAT0013387 25 0.74

mtr-miR2630b MIMAT0013371 25 0.74

mtr-miR2630g MIMAT0013379 25 0.74

mtr-miR2630c MIMAT0013372 25 0.74

mtr-miR2630i MIMAT0013381 25 0.74

mtr-miR2630v MIMAT0013394 25 0.74

mtr-miR2630l MIMAT0013384 25 0.74

mtr-miR2630u MIMAT0013393 25 0.74

mtr-miR2630d MIMAT0013376 25 0.74

mtr-miR2630r MIMAT0013390 25 0.74

mtr-miR2630t MIMAT0013392 25 0.74

mtr-miR2630e MIMAT0013377 25 0.74

mtr-miR2630q MIMAT0013389 25 0.74

osa-miR1853-5p MIMAT0007773 23 2.6

mtr-miR2657a MIMAT0013457 23 2.6

mtr-miR2657b MIMAT0013458 23 2.6

ath-miR1888 MIMAT0007855 23 2.6

aly-miR166e* MIMAT0017467 23 2.6

aly-miR3438-3p MIMAT0017707 23 2.6

zma-miR169m* MIMAT0015324 21 9.1

zma-miR164e MIMAT0013982 21 9.1

zma-miR166g* MIMAT0015150 21 9.1

zma-miR166b* MIMAT0015151 21 9.1

zma-miR166d* MIMAT0015153 21 9.1

zma-miR166a* MIMAT0015146 21 9.1

zma-miR166m* MIMAT0015204 21 9.1

zma-miR166c* MIMAT0015152 21 9.1

sbi-miR164c MIMAT0001754 21 9.1

osa-miR2865 MIMAT0013812 21 9.1

csi-miR166e* MIMAT0017386 21 9.1

mtr-miR2642 MIMAT0013410 21 9.1

mtr-miR2611 MIMAT0013320 21 9.1

gso-miR3522a MIMAT0016359 21 9.1

gso-miR3522b MIMAT0016360 21 9.1

gma-miR4376 MIMAT0018280 21 9.1

bra-miR824 MIMAT0005598 21 9.1

bol-miR824 MIMAT0005597 21 9.1

bna-miR824 MIMAT0005599 21 9.1

ath-miR824 MIMAT0004277 21 9.1

ath-miR774 MIMAT0003933 21 9.1

aly-miR165a* MIMAT0017455 21 9.1

aly-miR166a* MIMAT0017459 21 9.1

aly-miR166c* MIMAT0017463 21 9.1

aly-miR168a* MIMAT0017482 21 9.1

aly-miR166d* MIMAT0017465 21 9.1

aly-miR824 MIMAT0017603 21 9.1

ppt-miR166m MIMAT0005049 21 9.1

ppt-miR414 MIMAT0004357 21 9.1

>mtr-miR2630n MIMAT0013386

Length = 21

Score = 24.7 bits (26), Expect = 0.74

Identities = 13/13 (100%)

Strand = Plus / Minus

Query: 1152 caaggaccaaaac 1164

|||||||||||||

Sbjct: 15 caaggaccaaaac 3

>mtr-miR2630y MIMAT0013375

Length = 21

Score = 24.7 bits (26), Expect = 0.74

Identities = 13/13 (100%)

Strand = Plus / Minus

Query: 1152 caaggaccaaaac 1164

|||||||||||||

Sbjct: 15 caaggaccaaaac 3

>mtr-miR2630a MIMAT0013370

Length = 21

Score = 24.7 bits (26), Expect = 0.74

Identities = 13/13 (100%)

Strand = Plus / Minus

Query: 1152 caaggaccaaaac 1164

|||||||||||||

Sbjct: 15 caaggaccaaaac 3

>mtr-miR2630k MIMAT0013383

Length = 21

Score = 24.7 bits (26), Expect = 0.74

Identities = 13/13 (100%)

Strand = Plus / Minus

Query: 1152 caaggaccaaaac 1164

|||||||||||||

Sbjct: 15 caaggaccaaaac 3

>mtr-miR2630p MIMAT0013388

Length = 21

Score = 24.7 bits (26), Expect = 0.74

Identities = 13/13 (100%)

Strand = Plus / Minus

Query: 1152 caaggaccaaaac 1164

|||||||||||||

Sbjct: 15 caaggaccaaaac 3

>mtr-miR2630x MIMAT0013374

Length = 21

Score = 24.7 bits (26), Expect = 0.74

Identities = 13/13 (100%)

Strand = Plus / Minus

Query: 1152 caaggaccaaaac 1164

|||||||||||||

Sbjct: 15 caaggaccaaaac 3

>mtr-miR2630h MIMAT0013380

Length = 21

Score = 24.7 bits (26), Expect = 0.74

Identities = 13/13 (100%)

Strand = Plus / Minus

Query: 1152 caaggaccaaaac 1164

|||||||||||||

Sbjct: 15 caaggaccaaaac 3

>mtr-miR2630m MIMAT0013385

Length = 21

Score = 24.7 bits (26), Expect = 0.74

Identities = 13/13 (100%)

Strand = Plus / Minus

Query: 1152 caaggaccaaaac 1164

|||||||||||||

Sbjct: 15 caaggaccaaaac 3

>mtr-miR2630w MIMAT0013373

Length = 21

Score = 24.7 bits (26), Expect = 0.74

Identities = 13/13 (100%)

Strand = Plus / Minus

Query: 1152 caaggaccaaaac 1164

|||||||||||||

Sbjct: 15 caaggaccaaaac 3

>mtr-miR2630f MIMAT0013378

Length = 21

Score = 24.7 bits (26), Expect = 0.74

Identities = 13/13 (100%)

Strand = Plus / Minus

Query: 1152 caaggaccaaaac 1164

|||||||||||||

Sbjct: 15 caaggaccaaaac 3

>mtr-miR2630s MIMAT0013391

Length = 21

Score = 24.7 bits (26), Expect = 0.74

Identities = 13/13 (100%)

Strand = Plus / Minus

Query: 1152 caaggaccaaaac 1164

|||||||||||||

Sbjct: 15 caaggaccaaaac 3

>mtr-miR2630j MIMAT0013382

Length = 21

Score = 24.7 bits (26), Expect = 0.74

Identities = 13/13 (100%)

Strand = Plus / Minus

Query: 1152 caaggaccaaaac 1164

|||||||||||||

Sbjct: 15 caaggaccaaaac 3

>mtr-miR2630o MIMAT0013387

Length = 21

Score = 24.7 bits (26), Expect = 0.74

Identities = 13/13 (100%)

Strand = Plus / Minus

Query: 1152 caaggaccaaaac 1164

|||||||||||||

Sbjct: 15 caaggaccaaaac 3

>mtr-miR2630b MIMAT0013371

Length = 21

Score = 24.7 bits (26), Expect = 0.74

Identities = 13/13 (100%)

Strand = Plus / Minus

Query: 1152 caaggaccaaaac 1164

|||||||||||||

Sbjct: 15 caaggaccaaaac 3

>mtr-miR2630g MIMAT0013379

Length = 21

Score = 24.7 bits (26), Expect = 0.74

Identities = 13/13 (100%)

Strand = Plus / Minus

Query: 1152 caaggaccaaaac 1164

|||||||||||||

Sbjct: 15 caaggaccaaaac 3

>mtr-miR2630c MIMAT0013372

Length = 21

Score = 24.7 bits (26), Expect = 0.74

Identities = 13/13 (100%)

Strand = Plus / Minus

Query: 1152 caaggaccaaaac 1164

|||||||||||||

Sbjct: 15 caaggaccaaaac 3

>mtr-miR2630i MIMAT0013381

Length = 21

Score = 24.7 bits (26), Expect = 0.74

Identities = 13/13 (100%)

Strand = Plus / Minus

Query: 1152 caaggaccaaaac 1164

|||||||||||||

Sbjct: 15 caaggaccaaaac 3

>mtr-miR2630v MIMAT0013394

Length = 21

Score = 24.7 bits (26), Expect = 0.74

Identities = 13/13 (100%)

Strand = Plus / Minus

Query: 1152 caaggaccaaaac 1164

|||||||||||||

Sbjct: 15 caaggaccaaaac 3

>mtr-miR2630l MIMAT0013384

Length = 21

Score = 24.7 bits (26), Expect = 0.74

Identities = 13/13 (100%)

Strand = Plus / Minus

Query: 1152 caaggaccaaaac 1164

|||||||||||||

Sbjct: 15 caaggaccaaaac 3

>mtr-miR2630u MIMAT0013393

Length = 21

Score = 24.7 bits (26), Expect = 0.74

Identities = 13/13 (100%)

Strand = Plus / Minus

Query: 1152 caaggaccaaaac 1164

|||||||||||||

Sbjct: 15 caaggaccaaaac 3

>mtr-miR2630d MIMAT0013376

Length = 21

Score = 24.7 bits (26), Expect = 0.74

Identities = 13/13 (100%)

Strand = Plus / Minus

Query: 1152 caaggaccaaaac 1164

|||||||||||||

Sbjct: 15 caaggaccaaaac 3

>mtr-miR2630r MIMAT0013390

Length = 21

Score = 24.7 bits (26), Expect = 0.74

Identities = 13/13 (100%)

Strand = Plus / Minus

Query: 1152 caaggaccaaaac 1164

|||||||||||||

Sbjct: 15 caaggaccaaaac 3

>mtr-miR2630t MIMAT0013392

Length = 21

Score = 24.7 bits (26), Expect = 0.74

Identities = 13/13 (100%)

Strand = Plus / Minus

Query: 1152 caaggaccaaaac 1164

|||||||||||||

Sbjct: 15 caaggaccaaaac 3

>mtr-miR2630e MIMAT0013377

Length = 21

Score = 24.7 bits (26), Expect = 0.74

Identities = 13/13 (100%)

Strand = Plus / Minus

Query: 1152 caaggaccaaaac 1164

|||||||||||||

Sbjct: 15 caaggaccaaaac 3

>mtr-miR2630q MIMAT0013389

Length = 21

Score = 24.7 bits (26), Expect = 0.74

Identities = 13/13 (100%)

Strand = Plus / Minus

Query: 1152 caaggaccaaaac 1164

|||||||||||||

Sbjct: 15 caaggaccaaaac 3

>osa-miR1853-5p MIMAT0007773

Length = 24

Score = 22.9 bits (24), Expect = 2.6

Identities = 12/12 (100%)

Strand = Plus / Plus

Query: 735 aacattcccaat 746

||||||||||||

Sbjct: 9 aacattcccaat 20

Score = 22.9 bits (24), Expect = 2.6

Identities = 12/12 (100%)

Strand = Plus / Plus

Query: 735 aacattcccaat 746

||||||||||||

Sbjct: 9 aacattcccaat 20

>mtr-miR2657a MIMAT0013457

Length = 22

Score = 22.9 bits (24), Expect = 2.6

Identities = 14/15 (93%)

Strand = Plus / Plus

Query: 1387 ttatttgatcgattt 1401

|||||| ||||||||

Sbjct: 3 ttatttcatcgattt 17

>mtr-miR2657b MIMAT0013458

Length = 22

Score = 22.9 bits (24), Expect = 2.6

Identities = 14/15 (93%)

Strand = Plus / Plus

Query: 1387 ttatttgatcgattt 1401

|||||| ||||||||

Sbjct: 3 ttatttcatcgattt 17

>ath-miR1888 MIMAT0007855

Length = 21

Score = 22.9 bits (24), Expect = 2.6

Identities = 12/12 (100%)

Strand = Plus / Minus

Query: 79 ttcttcacaaat 90

||||||||||||

Sbjct: 21 ttcttcacaaat 10

>aly-miR166e* MIMAT0017467

Length = 21

Score = 22.9 bits (24), Expect = 2.6

Identities = 17/20 (85%)

Strand = Plus / Minus

Query: 723 ctctttccaggcaacattcc 742

||| | |||| |||||||||

Sbjct: 20 ctcgtgccagacaacattcc 1

>aly-miR3438-3p MIMAT0017707

Length = 21

Score = 22.9 bits (24), Expect = 2.6

Identities = 12/12 (100%)

Strand = Plus / Plus

Query: 1166 gcttcatctctg 1177

||||||||||||

Sbjct: 7 gcttcatctctg 18

>zma-miR169m* MIMAT0015324

Length = 20

Score = 21.1 bits (22), Expect = 9.1

Identities = 11/11 (100%)

Strand = Plus / Minus

Query: 203 caagaatggat 213

|||||||||||

Sbjct: 14 caagaatggat 4

>zma-miR164e MIMAT0013982

Length = 21

Score = 21.1 bits (22), Expect = 9.1

Identities = 11/11 (100%)

Strand = Plus / Minus

Query: 692 tgtcctgcttc 702

|||||||||||

Sbjct: 15 tgtcctgcttc 5

>zma-miR166g* MIMAT0015150

Length = 21

Score = 21.1 bits (22), Expect = 9.1

Identities = 13/14 (92%)

Strand = Plus / Minus

Query: 729 ccaggcaacattcc 742

|||| |||||||||

Sbjct: 14 ccagacaacattcc 1

>zma-miR166b* MIMAT0015151

Length = 21

Score = 21.1 bits (22), Expect = 9.1

Identities = 13/14 (92%)

Strand = Plus / Minus

Query: 729 ccaggcaacattcc 742

|||| |||||||||

Sbjct: 14 ccagacaacattcc 1

>zma-miR166d* MIMAT0015153

Length = 21

Score = 21.1 bits (22), Expect = 9.1

Identities = 13/14 (92%)

Strand = Plus / Minus

Query: 729 ccaggcaacattcc 742

|||| |||||||||

Sbjct: 14 ccagacaacattcc 1

>zma-miR166a* MIMAT0015146

Length = 21

Score = 21.1 bits (22), Expect = 9.1

Identities = 13/14 (92%)

Strand = Plus / Minus

Query: 729 ccaggcaacattcc 742

|||| |||||||||

Sbjct: 14 ccagacaacattcc 1

>zma-miR166m* MIMAT0015204

Length = 21

Score = 21.1 bits (22), Expect = 9.1

Identities = 13/14 (92%)

Strand = Plus / Minus

Query: 729 ccaggcaacattcc 742

|||| |||||||||

Sbjct: 14 ccagccaacattcc 1

>zma-miR166c* MIMAT0015152

Length = 21

Score = 21.1 bits (22), Expect = 9.1

Identities = 13/14 (92%)

Strand = Plus / Minus

Query: 729 ccaggcaacattcc 742

|||| |||||||||

Sbjct: 14 ccagacaacattcc 1

>sbi-miR164c MIMAT0001754

Length = 21

Score = 21.1 bits (22), Expect = 9.1

Identities = 11/11 (100%)

Strand = Plus / Minus

Query: 692 tgtcctgcttc 702

|||||||||||

Sbjct: 15 tgtcctgcttc 5

>osa-miR2865 MIMAT0013812

Length = 22

Score = 21.1 bits (22), Expect = 9.1

Identities = 11/11 (100%)

Strand = Plus / Plus

Query: 255 gtcgactgtac 265

|||||||||||

Sbjct: 8 gtcgactgtac 18

Score = 21.1 bits (22), Expect = 9.1

Identities = 11/11 (100%)

Strand = Plus / Plus

Query: 255 gtcgactgtac 265

|||||||||||

Sbjct: 8 gtcgactgtac 18

>csi-miR166e* MIMAT0017386

Length = 21

Score = 21.1 bits (22), Expect = 9.1

Identities = 13/14 (92%)

Strand = Plus / Minus

Query: 729 ccaggcaacattcc 742

|||| |||||||||

Sbjct: 14 ccagacaacattcc 1

>mtr-miR2642 MIMAT0013410

Length = 21

Score = 21.1 bits (22), Expect = 9.1

Identities = 11/11 (100%)

Strand = Plus / Plus

Query: 572 gagtttcatca 582

|||||||||||

Sbjct: 3 gagtttcatca 13

>mtr-miR2611 MIMAT0013320

Length = 21

Score = 21.1 bits (22), Expect = 9.1

Identities = 11/11 (100%)

Strand = Plus / Minus

Query: 264 actgacaaata 274

|||||||||||

Sbjct: 11 actgacaaata 1

>gso-miR3522a MIMAT0016359

Length = 21

Score = 21.1 bits (22), Expect = 9.1

Identities = 11/11 (100%)

Strand = Plus / Plus

Query: 1313 ccaaatgagca 1323

|||||||||||

Sbjct: 6 ccaaatgagca 16

>gso-miR3522b MIMAT0016360

Length = 22

Score = 21.1 bits (22), Expect = 9.1

Identities = 11/11 (100%)

Strand = Plus / Plus

Query: 1313 ccaaatgagca 1323

|||||||||||

Sbjct: 6 ccaaatgagca 16

>gma-miR4376 MIMAT0018280

Length = 22

Score = 21.1 bits (22), Expect = 9.1

Identities = 16/19 (84%)

Strand = Plus / Minus

Query: 1163 acggcttcatctctgctgc 1181

|| || |||||||| ||||

Sbjct: 22 acagcgtcatctctcctgc 4

>bra-miR824 MIMAT0005598

Length = 21

Score = 21.1 bits (22), Expect = 9.1

Identities = 15/16 (93%), Gaps = 1/16 (6%)

Strand = Plus / Minus

Query: 78 cttcttcacaaatggt 93

||||| ||||||||||

Sbjct: 18 cttct-cacaaatggt 4

>bol-miR824 MIMAT0005597

Length = 21

Score = 21.1 bits (22), Expect = 9.1

Identities = 15/16 (93%), Gaps = 1/16 (6%)

Strand = Plus / Minus

Query: 78 cttcttcacaaatggt 93

||||| ||||||||||

Sbjct: 18 cttct-cacaaatggt 4

>bna-miR824 MIMAT0005599

Length = 21

Score = 21.1 bits (22), Expect = 9.1

Identities = 15/16 (93%), Gaps = 1/16 (6%)

Strand = Plus / Minus

Query: 78 cttcttcacaaatggt 93

||||| ||||||||||

Sbjct: 18 cttct-cacaaatggt 4

>ath-miR824 MIMAT0004277

Length = 21

Score = 21.1 bits (22), Expect = 9.1

Identities = 15/16 (93%), Gaps = 1/16 (6%)

Strand = Plus / Minus

Query: 78 cttcttcacaaatggt 93

||||| ||||||||||

Sbjct: 18 cttct-cacaaatggt 4

>ath-miR774 MIMAT0003933

Length = 21

Score = 21.1 bits (22), Expect = 9.1

Identities = 17/21 (80%)

Strand = Plus / Minus

Query: 1308 gatggccaaatgagcacccaa 1328

|||||||| ||| | | ||||

Sbjct: 21 gatggccatatgggtaaccaa 1

>aly-miR165a* MIMAT0017455

Length = 20

Score = 21.1 bits (22), Expect = 9.1

Identities = 13/14 (92%)

Strand = Plus / Minus

Query: 728 tccaggcaacattc 741

||||| ||||||||

Sbjct: 14 tccagacaacattc 1

>aly-miR166a* MIMAT0017459

Length = 21

Score = 21.1 bits (22), Expect = 9.1

Identities = 13/14 (92%)

Strand = Plus / Minus

Query: 729 ccaggcaacattcc 742

|||| |||||||||

Sbjct: 14 ccagacaacattcc 1

>aly-miR166c* MIMAT0017463

Length = 21

Score = 21.1 bits (22), Expect = 9.1

Identities = 13/14 (92%)

Strand = Plus / Minus

Query: 729 ccaggcaacattcc 742

|||| |||||||||

Sbjct: 14 ccagacaacattcc 1

>aly-miR168a* MIMAT0017482

Length = 21

Score = 21.1 bits (22), Expect = 9.1

Identities = 13/14 (92%)

Strand = Plus / Minus

Query: 608 attcagtagatgca 621

||||||| ||||||

Sbjct: 21 attcagttgatgca 8

>aly-miR166d* MIMAT0017465

Length = 21

Score = 21.1 bits (22), Expect = 9.1

Identities = 13/14 (92%)

Strand = Plus / Minus

Query: 729 ccaggcaacattcc 742

|||| |||||||||

Sbjct: 14 ccagacaacattcc 1

>aly-miR824 MIMAT0017603

Length = 21

Score = 21.1 bits (22), Expect = 9.1

Identities = 15/16 (93%), Gaps = 1/16 (6%)

Strand = Plus / Minus

Query: 78 cttcttcacaaatggt 93

||||| ||||||||||

Sbjct: 18 cttct-cacaaatggt 4

>ppt-miR166m MIMAT0005049

Length = 21

Score = 21.1 bits (22), Expect = 9.1

Identities = 13/14 (92%)

Strand = Plus / Plus

Query: 729 ccaggcaacattcc 742

||||||| ||||||

Sbjct: 6 ccaggcatcattcc 19

>ppt-miR414 MIMAT0004357

Length = 21

Score = 21.1 bits (22), Expect = 9.1

Identities = 11/11 (100%)

Strand = Plus / Plus

Query: 793 catcctcgtcc 803

|||||||||||

Sbjct: 11 catcctcgtcc 21

BLASTN 2.2.21 [Jun-14-2009]

Reference: Altschul, Stephen F., Thomas L. Madden, Alejandro A. Schaffer,

Jinghui Zhang, Zheng Zhang, Webb Miller, and David J. Lipman (1997),

"Gapped BLAST and PSI-BLAST: a new generation of protein database search

programs", Nucleic Acids Res. 25:3389-3402.

Query= gi|1755004|gb|U49103.1|TAU49103 Triticum aestivum calmodulin

TaCaM3-2 mRNA, complete cds

(811 letters)

Database: matureREL16.txt

4142 sequences; 88,243 total letters

Searching..................................................done

Score E

Sequences producing significant alignments: (bits) Value

mtr-miR2630n MIMAT0013386 25 0.51

mtr-miR2630y MIMAT0013375 25 0.51

mtr-miR2630a MIMAT0013370 25 0.51

mtr-miR2630k MIMAT0013383 25 0.51

mtr-miR2630p MIMAT0013388 25 0.51

mtr-miR2630x MIMAT0013374 25 0.51

mtr-miR2630h MIMAT0013380 25 0.51

mtr-miR2630m MIMAT0013385 25 0.51

mtr-miR2630w MIMAT0013373 25 0.51

mtr-miR2630f MIMAT0013378 25 0.51

mtr-miR2630s MIMAT0013391 25 0.51

mtr-miR2630j MIMAT0013382 25 0.51

mtr-miR2630o MIMAT0013387 25 0.51

mtr-miR2630b MIMAT0013371 25 0.51

mtr-miR2630g MIMAT0013379 25 0.51

mtr-miR2630c MIMAT0013372 25 0.51

mtr-miR2630i MIMAT0013381 25 0.51

mtr-miR2630v MIMAT0013394 25 0.51

mtr-miR2630l MIMAT0013384 25 0.51

mtr-miR2630u MIMAT0013393 25 0.51

mtr-miR2630d MIMAT0013376 25 0.51

mtr-miR2630r MIMAT0013390 25 0.51

mtr-miR2630t MIMAT0013392 25 0.51

mtr-miR2630e MIMAT0013377 25 0.51

mtr-miR2630q MIMAT0013389 25 0.51

zma-miR1432 MIMAT0014032 23 1.8

sbi-miR1432 MIMAT0011390 23 1.8

osa-miR1318 MIMAT0009135 23 1.8

osa-miR1432 MIMAT0005966 23 1.8

osa-miR1318 MIMAT0009135 23 1.8

osa-miR1432 MIMAT0005966 23 1.8

mtr-miR2657a MIMAT0013457 23 1.8

mtr-miR2657b MIMAT0013458 23 1.8

aly-miR831 MIMAT0017614 21 6.2

>mtr-miR2630n MIMAT0013386

Length = 21

Score = 24.7 bits (26), Expect = 0.51

Identities = 13/13 (100%)

Strand = Plus / Minus

Query: 384 caaggaccaaaac 396

|||||||||||||

Sbjct: 15 caaggaccaaaac 3

>mtr-miR2630y MIMAT0013375

Length = 21

Score = 24.7 bits (26), Expect = 0.51

Identities = 13/13 (100%)

Strand = Plus / Minus

Query: 384 caaggaccaaaac 396

|||||||||||||

Sbjct: 15 caaggaccaaaac 3

>mtr-miR2630a MIMAT0013370

Length = 21

Score = 24.7 bits (26), Expect = 0.51

Identities = 13/13 (100%)

Strand = Plus / Minus

Query: 384 caaggaccaaaac 396

|||||||||||||

Sbjct: 15 caaggaccaaaac 3

>mtr-miR2630k MIMAT0013383

Length = 21

Score = 24.7 bits (26), Expect = 0.51

Identities = 13/13 (100%)

Strand = Plus / Minus

Query: 384 caaggaccaaaac 396

|||||||||||||

Sbjct: 15 caaggaccaaaac 3

>mtr-miR2630p MIMAT0013388

Length = 21

Score = 24.7 bits (26), Expect = 0.51

Identities = 13/13 (100%)

Strand = Plus / Minus

Query: 384 caaggaccaaaac 396

|||||||||||||

Sbjct: 15 caaggaccaaaac 3

>mtr-miR2630x MIMAT0013374

Length = 21

Score = 24.7 bits (26), Expect = 0.51

Identities = 13/13 (100%)

Strand = Plus / Minus

Query: 384 caaggaccaaaac 396

|||||||||||||

Sbjct: 15 caaggaccaaaac 3

>mtr-miR2630h MIMAT0013380

Length = 21

Score = 24.7 bits (26), Expect = 0.51

Identities = 13/13 (100%)

Strand = Plus / Minus

Query: 384 caaggaccaaaac 396

|||||||||||||

Sbjct: 15 caaggaccaaaac 3

>mtr-miR2630m MIMAT0013385

Length = 21

Score = 24.7 bits (26), Expect = 0.51

Identities = 13/13 (100%)

Strand = Plus / Minus

Query: 384 caaggaccaaaac 396

|||||||||||||

Sbjct: 15 caaggaccaaaac 3

>mtr-miR2630w MIMAT0013373

Length = 21

Score = 24.7 bits (26), Expect = 0.51

Identities = 13/13 (100%)

Strand = Plus / Minus

Query: 384 caaggaccaaaac 396

|||||||||||||

Sbjct: 15 caaggaccaaaac 3

>mtr-miR2630f MIMAT0013378

Length = 21

Score = 24.7 bits (26), Expect = 0.51

Identities = 13/13 (100%)

Strand = Plus / Minus

Query: 384 caaggaccaaaac 396

|||||||||||||

Sbjct: 15 caaggaccaaaac 3

>mtr-miR2630s MIMAT0013391

Length = 21

Score = 24.7 bits (26), Expect = 0.51

Identities = 13/13 (100%)

Strand = Plus / Minus

Query: 384 caaggaccaaaac 396

|||||||||||||

Sbjct: 15 caaggaccaaaac 3

>mtr-miR2630j MIMAT0013382

Length = 21

Score = 24.7 bits (26), Expect = 0.51

Identities = 13/13 (100%)

Strand = Plus / Minus

Query: 384 caaggaccaaaac 396

|||||||||||||

Sbjct: 15 caaggaccaaaac 3

>mtr-miR2630o MIMAT0013387

Length = 21

Score = 24.7 bits (26), Expect = 0.51

Identities = 13/13 (100%)

Strand = Plus / Minus

Query: 384 caaggaccaaaac 396

|||||||||||||

Sbjct: 15 caaggaccaaaac 3

>mtr-miR2630b MIMAT0013371

Length = 21

Score = 24.7 bits (26), Expect = 0.51

Identities = 13/13 (100%)

Strand = Plus / Minus

Query: 384 caaggaccaaaac 396

|||||||||||||

Sbjct: 15 caaggaccaaaac 3

>mtr-miR2630g MIMAT0013379

Length = 21

Score = 24.7 bits (26), Expect = 0.51

Identities = 13/13 (100%)

Strand = Plus / Minus

Query: 384 caaggaccaaaac 396

|||||||||||||

Sbjct: 15 caaggaccaaaac 3

>mtr-miR2630c MIMAT0013372

Length = 21

Score = 24.7 bits (26), Expect = 0.51

Identities = 13/13 (100%)

Strand = Plus / Minus

Query: 384 caaggaccaaaac 396

|||||||||||||

Sbjct: 15 caaggaccaaaac 3

>mtr-miR2630i MIMAT0013381

Length = 21

Score = 24.7 bits (26), Expect = 0.51

Identities = 13/13 (100%)

Strand = Plus / Minus

Query: 384 caaggaccaaaac 396

|||||||||||||

Sbjct: 15 caaggaccaaaac 3

>mtr-miR2630v MIMAT0013394

Length = 21

Score = 24.7 bits (26), Expect = 0.51

Identities = 13/13 (100%)

Strand = Plus / Minus

Query: 384 caaggaccaaaac 396

|||||||||||||

Sbjct: 15 caaggaccaaaac 3

>mtr-miR2630l MIMAT0013384

Length = 21

Score = 24.7 bits (26), Expect = 0.51

Identities = 13/13 (100%)

Strand = Plus / Minus

Query: 384 caaggaccaaaac 396

|||||||||||||

Sbjct: 15 caaggaccaaaac 3

>mtr-miR2630u MIMAT0013393

Length = 21

Score = 24.7 bits (26), Expect = 0.51

Identities = 13/13 (100%)

Strand = Plus / Minus

Query: 384 caaggaccaaaac 396

|||||||||||||

Sbjct: 15 caaggaccaaaac 3

>mtr-miR2630d MIMAT0013376

Length = 21

Score = 24.7 bits (26), Expect = 0.51

Identities = 13/13 (100%)

Strand = Plus / Minus

Query: 384 caaggaccaaaac 396

|||||||||||||

Sbjct: 15 caaggaccaaaac 3

>mtr-miR2630r MIMAT0013390

Length = 21

Score = 24.7 bits (26), Expect = 0.51

Identities = 13/13 (100%)

Strand = Plus / Minus

Query: 384 caaggaccaaaac 396

|||||||||||||

Sbjct: 15 caaggaccaaaac 3

>mtr-miR2630t MIMAT0013392

Length = 21

Score = 24.7 bits (26), Expect = 0.51

Identities = 13/13 (100%)

Strand = Plus / Minus

Query: 384 caaggaccaaaac 396

|||||||||||||

Sbjct: 15 caaggaccaaaac 3

>mtr-miR2630e MIMAT0013377

Length = 21

Score = 24.7 bits (26), Expect = 0.51

Identities = 13/13 (100%)

Strand = Plus / Minus

Query: 384 caaggaccaaaac 396

|||||||||||||

Sbjct: 15 caaggaccaaaac 3

>mtr-miR2630q MIMAT0013389

Length = 21

Score = 24.7 bits (26), Expect = 0.51

Identities = 13/13 (100%)

Strand = Plus / Minus

Query: 384 caaggaccaaaac 396

|||||||||||||

Sbjct: 15 caaggaccaaaac 3

>zma-miR1432 MIMAT0014032

Length = 21

Score = 22.9 bits (24), Expect = 1.8

Identities = 15/17 (88%)

Strand = Plus / Minus

Query: 396 cggtttcatctctgctg 412

|||| |||||||| |||

Sbjct: 19 cggtgtcatctctcctg 3

>sbi-miR1432 MIMAT0011390

Length = 21

Score = 22.9 bits (24), Expect = 1.8

Identities = 15/17 (88%)

Strand = Plus / Minus

Query: 396 cggtttcatctctgctg 412

|||| |||||||| |||

Sbjct: 19 cggtgtcatctctcctg 3

>osa-miR1318 MIMAT0009135

Length = 20

Score = 22.9 bits (24), Expect = 1.8

Identities = 15/17 (88%)

Strand = Plus / Minus

Query: 396 cggtttcatctctgctg 412

|||| |||||||| |||

Sbjct: 18 cggtgtcatctctcctg 2

>osa-miR1432 MIMAT0005966

Length = 21

Score = 22.9 bits (24), Expect = 1.8

Identities = 15/17 (88%)

Strand = Plus / Minus

Query: 396 cggtttcatctctgctg 412

|||| |||||||| |||

Sbjct: 19 cggtgtcatctctcctg 3

Score = 22.9 bits (24), Expect = 1.8

Identities = 15/17 (88%)

Strand = Plus / Minus

Query: 396 cggtttcatctctgctg 412

|||| |||||||| |||

Sbjct: 18 cggtgtcatctctcctg 2

Score = 22.9 bits (24), Expect = 1.8

Identities = 15/17 (88%)

Strand = Plus / Minus

Query: 396 cggtttcatctctgctg 412

|||| |||||||| |||

Sbjct: 19 cggtgtcatctctcctg 3

>mtr-miR2657a MIMAT0013457

Length = 22

Score = 22.9 bits (24), Expect = 1.8

Identities = 14/15 (93%)

Strand = Plus / Plus

Query: 621 ttatttgatcgattt 635

|||||| ||||||||

Sbjct: 3 ttatttcatcgattt 17

>mtr-miR2657b MIMAT0013458

Length = 22

Score = 22.9 bits (24), Expect = 1.8

Identities = 14/15 (93%)

Strand = Plus / Plus

Query: 621 ttatttgatcgattt 635

|||||| ||||||||

Sbjct: 3 ttatttcatcgattt 17

>aly-miR831 MIMAT0017614

Length = 22

Score = 21.1 bits (22), Expect = 6.2

Identities = 11/11 (100%)

Strand = Plus / Minus

Query: 517 tacgaggagtt 527

|||||||||||

Sbjct: 13 tacgaggagtt 3

BLASTN 2.2.21 [Jun-14-2009]

Reference: Altschul, Stephen F., Thomas L. Madden, Alejandro A. Schaffer,

Jinghui Zhang, Zheng Zhang, Webb Miller, and David J. Lipman (1997),

"Gapped BLAST and PSI-BLAST: a new generation of protein database search

programs", Nucleic Acids Res. 25:3389-3402.

Query= gi|1755006|gb|U49104.1|TAU49104 Triticum aestivum calmodulin

TaCaM3-3 mRNA, complete cds

(846 letters)

Database: matureREL16.txt

4142 sequences; 88,243 total letters

Searching..................................................done

Score E

Sequences producing significant alignments: (bits) Value

mtr-miR2630n MIMAT0013386 25 0.53

mtr-miR2630y MIMAT0013375 25 0.53

mtr-miR2630a MIMAT0013370 25 0.53

mtr-miR2630k MIMAT0013383 25 0.53

mtr-miR2630p MIMAT0013388 25 0.53

mtr-miR2630x MIMAT0013374 25 0.53

mtr-miR2630h MIMAT0013380 25 0.53

mtr-miR2630m MIMAT0013385 25 0.53

mtr-miR2630w MIMAT0013373 25 0.53

mtr-miR2630f MIMAT0013378 25 0.53

mtr-miR2630s MIMAT0013391 25 0.53

mtr-miR2630j MIMAT0013382 25 0.53

mtr-miR2630o MIMAT0013387 25 0.53

mtr-miR2630b MIMAT0013371 25 0.53

mtr-miR2630g MIMAT0013379 25 0.53

mtr-miR2630c MIMAT0013372 25 0.53

mtr-miR2630i MIMAT0013381 25 0.53

mtr-miR2630v MIMAT0013394 25 0.53

mtr-miR2630l MIMAT0013384 25 0.53

mtr-miR2630u MIMAT0013393 25 0.53

mtr-miR2630d MIMAT0013376 25 0.53

mtr-miR2630r MIMAT0013390 25 0.53

mtr-miR2630t MIMAT0013392 25 0.53

mtr-miR2630e MIMAT0013377 25 0.53

mtr-miR2630q MIMAT0013389 25 0.53

mtr-miR2657a MIMAT0013457 23 1.9

mtr-miR2657b MIMAT0013458 23 1.9

aly-miR3438-3p MIMAT0017707 23 1.9

gso-miR3522a MIMAT0016359 21 6.5

gso-miR3522b MIMAT0016360 21 6.5

gma-miR4376 MIMAT0018280 21 6.5

aly-miR831 MIMAT0017614 21 6.5

>mtr-miR2630n MIMAT0013386

Length = 21

Score = 24.7 bits (26), Expect = 0.53

Identities = 13/13 (100%)

Strand = Plus / Minus

Query: 386 caaggaccaaaac 398

|||||||||||||

Sbjct: 15 caaggaccaaaac 3

>mtr-miR2630y MIMAT0013375

Length = 21

Score = 24.7 bits (26), Expect = 0.53

Identities = 13/13 (100%)

Strand = Plus / Minus

Query: 386 caaggaccaaaac 398

|||||||||||||

Sbjct: 15 caaggaccaaaac 3

>mtr-miR2630a MIMAT0013370

Length = 21

Score = 24.7 bits (26), Expect = 0.53

Identities = 13/13 (100%)

Strand = Plus / Minus

Query: 386 caaggaccaaaac 398

|||||||||||||

Sbjct: 15 caaggaccaaaac 3

>mtr-miR2630k MIMAT0013383

Length = 21

Score = 24.7 bits (26), Expect = 0.53

Identities = 13/13 (100%)

Strand = Plus / Minus

Query: 386 caaggaccaaaac 398

|||||||||||||

Sbjct: 15 caaggaccaaaac 3

>mtr-miR2630p MIMAT0013388

Length = 21

Score = 24.7 bits (26), Expect = 0.53

Identities = 13/13 (100%)

Strand = Plus / Minus

Query: 386 caaggaccaaaac 398

|||||||||||||

Sbjct: 15 caaggaccaaaac 3

>mtr-miR2630x MIMAT0013374

Length = 21

Score = 24.7 bits (26), Expect = 0.53

Identities = 13/13 (100%)

Strand = Plus / Minus

Query: 386 caaggaccaaaac 398

|||||||||||||

Sbjct: 15 caaggaccaaaac 3

>mtr-miR2630h MIMAT0013380

Length = 21

Score = 24.7 bits (26), Expect = 0.53

Identities = 13/13 (100%)

Strand = Plus / Minus

Query: 386 caaggaccaaaac 398

|||||||||||||

Sbjct: 15 caaggaccaaaac 3

>mtr-miR2630m MIMAT0013385

Length = 21

Score = 24.7 bits (26), Expect = 0.53

Identities = 13/13 (100%)

Strand = Plus / Minus

Query: 386 caaggaccaaaac 398

|||||||||||||

Sbjct: 15 caaggaccaaaac 3

>mtr-miR2630w MIMAT0013373

Length = 21

Score = 24.7 bits (26), Expect = 0.53

Identities = 13/13 (100%)

Strand = Plus / Minus

Query: 386 caaggaccaaaac 398

|||||||||||||

Sbjct: 15 caaggaccaaaac 3

>mtr-miR2630f MIMAT0013378

Length = 21

Score = 24.7 bits (26), Expect = 0.53

Identities = 13/13 (100%)

Strand = Plus / Minus

Query: 386 caaggaccaaaac 398

|||||||||||||

Sbjct: 15 caaggaccaaaac 3

>mtr-miR2630s MIMAT0013391

Length = 21

Score = 24.7 bits (26), Expect = 0.53

Identities = 13/13 (100%)

Strand = Plus / Minus

Query: 386 caaggaccaaaac 398

|||||||||||||

Sbjct: 15 caaggaccaaaac 3

>mtr-miR2630j MIMAT0013382

Length = 21

Score = 24.7 bits (26), Expect = 0.53

Identities = 13/13 (100%)

Strand = Plus / Minus

Query: 386 caaggaccaaaac 398

|||||||||||||

Sbjct: 15 caaggaccaaaac 3

>mtr-miR2630o MIMAT0013387

Length = 21

Score = 24.7 bits (26), Expect = 0.53

Identities = 13/13 (100%)

Strand = Plus / Minus

Query: 386 caaggaccaaaac 398

|||||||||||||

Sbjct: 15 caaggaccaaaac 3

>mtr-miR2630b MIMAT0013371

Length = 21

Score = 24.7 bits (26), Expect = 0.53

Identities = 13/13 (100%)

Strand = Plus / Minus

Query: 386 caaggaccaaaac 398

|||||||||||||

Sbjct: 15 caaggaccaaaac 3

>mtr-miR2630g MIMAT0013379

Length = 21

Score = 24.7 bits (26), Expect = 0.53

Identities = 13/13 (100%)

Strand = Plus / Minus

Query: 386 caaggaccaaaac 398

|||||||||||||

Sbjct: 15 caaggaccaaaac 3

>mtr-miR2630c MIMAT0013372

Length = 21

Score = 24.7 bits (26), Expect = 0.53

Identities = 13/13 (100%)

Strand = Plus / Minus

Query: 386 caaggaccaaaac 398

|||||||||||||

Sbjct: 15 caaggaccaaaac 3

>mtr-miR2630i MIMAT0013381

Length = 21

Score = 24.7 bits (26), Expect = 0.53

Identities = 13/13 (100%)

Strand = Plus / Minus

Query: 386 caaggaccaaaac 398

|||||||||||||

Sbjct: 15 caaggaccaaaac 3

>mtr-miR2630v MIMAT0013394

Length = 21

Score = 24.7 bits (26), Expect = 0.53

Identities = 13/13 (100%)

Strand = Plus / Minus

Query: 386 caaggaccaaaac 398

|||||||||||||

Sbjct: 15 caaggaccaaaac 3

>mtr-miR2630l MIMAT0013384

Length = 21

Score = 24.7 bits (26), Expect = 0.53

Identities = 13/13 (100%)

Strand = Plus / Minus

Query: 386 caaggaccaaaac 398

|||||||||||||

Sbjct: 15 caaggaccaaaac 3

>mtr-miR2630u MIMAT0013393

Length = 21

Score = 24.7 bits (26), Expect = 0.53

Identities = 13/13 (100%)

Strand = Plus / Minus

Query: 386 caaggaccaaaac 398

|||||||||||||

Sbjct: 15 caaggaccaaaac 3

>mtr-miR2630d MIMAT0013376

Length = 21

Score = 24.7 bits (26), Expect = 0.53

Identities = 13/13 (100%)

Strand = Plus / Minus

Query: 386 caaggaccaaaac 398

|||||||||||||

Sbjct: 15 caaggaccaaaac 3

>mtr-miR2630r MIMAT0013390

Length = 21

Score = 24.7 bits (26), Expect = 0.53

Identities = 13/13 (100%)

Strand = Plus / Minus

Query: 386 caaggaccaaaac 398

|||||||||||||

Sbjct: 15 caaggaccaaaac 3

>mtr-miR2630t MIMAT0013392

Length = 21

Score = 24.7 bits (26), Expect = 0.53

Identities = 13/13 (100%)

Strand = Plus / Minus

Query: 386 caaggaccaaaac 398

|||||||||||||

Sbjct: 15 caaggaccaaaac 3

>mtr-miR2630e MIMAT0013377

Length = 21

Score = 24.7 bits (26), Expect = 0.53

Identities = 13/13 (100%)

Strand = Plus / Minus

Query: 386 caaggaccaaaac 398

|||||||||||||

Sbjct: 15 caaggaccaaaac 3

>mtr-miR2630q MIMAT0013389

Length = 21

Score = 24.7 bits (26), Expect = 0.53

Identities = 13/13 (100%)

Strand = Plus / Minus

Query: 386 caaggaccaaaac 398

|||||||||||||

Sbjct: 15 caaggaccaaaac 3

>mtr-miR2657a MIMAT0013457

Length = 22

Score = 22.9 bits (24), Expect = 1.9

Identities = 14/15 (93%)

Strand = Plus / Plus

Query: 622 ttatttgatcgattt 636

|||||| ||||||||

Sbjct: 3 ttatttcatcgattt 17

>mtr-miR2657b MIMAT0013458

Length = 22

Score = 22.9 bits (24), Expect = 1.9

Identities = 14/15 (93%)

Strand = Plus / Plus

Query: 622 ttatttgatcgattt 636

|||||| ||||||||

Sbjct: 3 ttatttcatcgattt 17

>aly-miR3438-3p MIMAT0017707

Length = 21

Score = 22.9 bits (24), Expect = 1.9

Identities = 12/12 (100%)

Strand = Plus / Plus

Query: 400 gcttcatctctg 411

||||||||||||

Sbjct: 7 gcttcatctctg 18

>gso-miR3522a MIMAT0016359

Length = 21

Score = 21.1 bits (22), Expect = 6.5

Identities = 11/11 (100%)

Strand = Plus / Plus

Query: 547 ccaaatgagca 557

|||||||||||

Sbjct: 6 ccaaatgagca 16

>gso-miR3522b MIMAT0016360

Length = 22

Score = 21.1 bits (22), Expect = 6.5

Identities = 11/11 (100%)

Strand = Plus / Plus

Query: 547 ccaaatgagca 557

|||||||||||

Sbjct: 6 ccaaatgagca 16

>gma-miR4376 MIMAT0018280

Length = 22

Score = 21.1 bits (22), Expect = 6.5

Identities = 16/19 (84%)

Strand = Plus / Minus

Query: 397 acggcttcatctctgctgc 415

|| || |||||||| ||||

Sbjct: 22 acagcgtcatctctcctgc 4

>aly-miR831 MIMAT0017614

Length = 22

Score = 21.1 bits (22), Expect = 6.5

Identities = 11/11 (100%)

Strand = Plus / Minus

Query: 519 tacgaggagtt 529

|||||||||||

Sbjct: 13 tacgaggagtt 3

BLASTN 2.2.21 [Jun-14-2009]

Reference: Altschul, Stephen F., Thomas L. Madden, Alejandro A. Schaffer,

Jinghui Zhang, Zheng Zhang, Webb Miller, and David J. Lipman (1997),

"Gapped BLAST and PSI-BLAST: a new generation of protein database search

programs", Nucleic Acids Res. 25:3389-3402.

Query= gi|1755008|gb|U49105.1|TAU49105 Triticum aestivum calmodulin

TaCaM4-1 mRNA, complete cds

(841 letters)

Database: matureREL16.txt

4142 sequences; 88,243 total letters

Searching..................................................done

Score E

Sequences producing significant alignments: (bits) Value

cre-miR1147.2 MIMAT0005385 25 0.53

zma-miR396c MIMAT0010076 23 1.8

zma-miR396d MIMAT0010077 23 1.8

sbi-miR396d MIMAT0011357 23 1.8

sbi-miR396e MIMAT0011358 23 1.8

osa-miR2096-3p MIMAT0010057 23 1.8

osa-miR396d MIMAT0001600 23 1.8

osa-miR396f MIMAT0010070 23 1.8

osa-miR396e MIMAT0001601 23 1.8

osa-miR2096-3p MIMAT0010057 23 1.8

osa-miR396d MIMAT0001600 23 1.8

osa-miR396f MIMAT0010070 23 1.8

osa-miR396e MIMAT0001601 23 1.8

far-miR396 MIMAT0018369 23 1.8

zma-miR160a* MIMAT0015132 21 6.4

osa-miR820b MIMAT0004080 21 6.4

osa-miR396g MIMAT0013833 21 6.4

osa-miR396i MIMAT0013835 21 6.4

osa-miR820c MIMAT0004081 21 6.4

osa-miR2118o MIMAT0011754 21 6.4

osa-miR820a MIMAT0004079 21 6.4

osa-miR396h MIMAT0013834 21 6.4

osa-miR820b MIMAT0004080 21 6.4

osa-miR396g MIMAT0013833 21 6.4

osa-miR396i MIMAT0013835 21 6.4

osa-miR820c MIMAT0004081 21 6.4

osa-miR2118o MIMAT0011754 21 6.4

osa-miR820a MIMAT0004079 21 6.4

osa-miR396h MIMAT0013834 21 6.4

ptc-miR396g MIMAT0002037 21 6.4

ptc-miR396f MIMAT0002036 21 6.4

gma-miR1533 MIMAT0007396 21 6.4

ahy-miR3510 MIMAT0016333 21 6.4

aly-miR831 MIMAT0017614 21 6.4

smo-miR1100 MIMAT0005256 21 6.4

pab-miR396c MIMAT0018154 21 6.4

pab-miR396b MIMAT0018153 21 6.4

>cre-miR1147.2 MIMAT0005385

Length = 20

Score = 24.7 bits (26), Expect = 0.53

Identities = 15/16 (93%)

Strand = Plus / Minus

Query: 795 tctgcaagacttggcc 810

||||| ||||||||||

Sbjct: 20 tctgccagacttggcc 5

>zma-miR396c MIMAT0010076

Length = 22

Score = 22.9 bits (24), Expect = 1.8

Identities = 14/15 (93%)

Strand = Plus / Minus

Query: 147 agttcaaggaagcct 161

|||||||| ||||||

Sbjct: 21 agttcaagaaagcct 7

>zma-miR396d MIMAT0010077

Length = 22

Score = 22.9 bits (24), Expect = 1.8

Identities = 14/15 (93%)

Strand = Plus / Minus

Query: 147 agttcaaggaagcct 161

|||||||| ||||||

Sbjct: 21 agttcaagaaagcct 7

>sbi-miR396d MIMAT0011357

Length = 22

Score = 22.9 bits (24), Expect = 1.8

Identities = 14/15 (93%)

Strand = Plus / Minus

Query: 147 agttcaaggaagcct 161

|||||||| ||||||

Sbjct: 21 agttcaagaaagcct 7

>sbi-miR396e MIMAT0011358

Length = 22

Score = 22.9 bits (24), Expect = 1.8

Identities = 14/15 (93%)

Strand = Plus / Minus

Query: 147 agttcaaggaagcct 161

|||||||| ||||||

Sbjct: 21 agttcaagaaagcct 7

>osa-miR2096-3p MIMAT0010057

Length = 21

Score = 22.9 bits (24), Expect = 1.8

Identities = 14/15 (93%)

Strand = Plus / Minus

Query: 563 tcccgccgatgtccc 577

|||||||||| ||||

Sbjct: 21 tcccgccgatttccc 7

>osa-miR396d MIMAT0001600

Length = 21

Score = 22.9 bits (24), Expect = 1.8

Identities = 14/15 (93%)

Strand = Plus / Minus

Query: 147 agttcaaggaagcct 161

|||||||| ||||||

Sbjct: 20 agttcaagaaagcct 6

>osa-miR396f MIMAT0010070

Length = 22

Score = 22.9 bits (24), Expect = 1.8

Identities = 14/15 (93%)

Strand = Plus / Minus

Query: 147 agttcaaggaagcct 161

|||||||| ||||||

Sbjct: 22 agttcaagaaagcct 8

>osa-miR396e MIMAT0001601

Length = 21

Score = 22.9 bits (24), Expect = 1.8

Identities = 14/15 (93%)

Strand = Plus / Minus

Query: 147 agttcaaggaagcct 161

|||||||| ||||||

Sbjct: 20 agttcaagaaagcct 6

Score = 22.9 bits (24), Expect = 1.8

Identities = 14/15 (93%)

Strand = Plus / Minus

Query: 563 tcccgccgatgtccc 577

|||||||||| ||||

Sbjct: 21 tcccgccgatttccc 7

Score = 22.9 bits (24), Expect = 1.8

Identities = 14/15 (93%)

Strand = Plus / Minus

Query: 147 agttcaaggaagcct 161

|||||||| ||||||

Sbjct: 20 agttcaagaaagcct 6

Score = 22.9 bits (24), Expect = 1.8

Identities = 14/15 (93%)

Strand = Plus / Minus

Query: 147 agttcaaggaagcct 161

|||||||| ||||||

Sbjct: 22 agttcaagaaagcct 8

Score = 22.9 bits (24), Expect = 1.8

Identities = 14/15 (93%)

Strand = Plus / Minus

Query: 147 agttcaaggaagcct 161

|||||||| ||||||

Sbjct: 20 agttcaagaaagcct 6

>far-miR396 MIMAT0018369

Length = 21

Score = 22.9 bits (24), Expect = 1.8

Identities = 14/15 (93%)

Strand = Plus / Minus

Query: 147 agttcaaggaagcct 161

|||||||| ||||||

Sbjct: 20 agttcaagaaagcct 6

>zma-miR160a* MIMAT0015132

Length = 21

Score = 21.1 bits (22), Expect = 6.4

Identities = 11/11 (100%)

Strand = Plus / Minus

Query: 804 cttggcccctt 814

|||||||||||

Sbjct: 17 cttggcccctt 7

>osa-miR820b MIMAT0004080

Length = 21

Score = 21.1 bits (22), Expect = 6.4

Identities = 13/14 (92%)

Strand = Plus / Minus

Query: 488 atccgcgaggccga 501

|||| |||||||||

Sbjct: 14 atccacgaggccga 1

>osa-miR396g MIMAT0013833

Length = 21

Score = 21.1 bits (22), Expect = 6.4

Identities = 13/14 (92%)

Strand = Plus / Minus

Query: 148 gttcaaggaagcct 161

||||||| ||||||

Sbjct: 19 gttcaagaaagcct 6

>osa-miR396i MIMAT0013835

Length = 21

Score = 21.1 bits (22), Expect = 6.4

Identities = 13/14 (92%)

Strand = Plus / Minus

Query: 148 gttcaaggaagcct 161

||||||| ||||||

Sbjct: 19 gttcaagaaagcct 6

>osa-miR820c MIMAT0004081

Length = 21

Score = 21.1 bits (22), Expect = 6.4

Identities = 13/14 (92%)

Strand = Plus / Minus

Query: 488 atccgcgaggccga 501

|||| |||||||||

Sbjct: 14 atccacgaggccga 1

>osa-miR2118o MIMAT0011754

Length = 22

Score = 21.1 bits (22), Expect = 6.4

Identities = 14/16 (87%)

Strand = Plus / Plus

Query: 325 cctgatggctcgcaag 340

||||||| ||| ||||

Sbjct: 3 cctgatgcctcccaag 18

>osa-miR820a MIMAT0004079

Length = 21

Score = 21.1 bits (22), Expect = 6.4

Identities = 13/14 (92%)

Strand = Plus / Minus

Query: 488 atccgcgaggccga 501

|||| |||||||||

Sbjct: 14 atccacgaggccga 1

>osa-miR396h MIMAT0013834

Length = 21

Score = 21.1 bits (22), Expect = 6.4

Identities = 13/14 (92%)

Strand = Plus / Minus

Query: 148 gttcaaggaagcct 161

||||||| ||||||

Sbjct: 19 gttcaagaaagcct 6

Score = 21.1 bits (22), Expect = 6.4

Identities = 13/14 (92%)

Strand = Plus / Minus

Query: 488 atccgcgaggccga 501

|||| |||||||||

Sbjct: 14 atccacgaggccga 1

Score = 21.1 bits (22), Expect = 6.4

Identities = 13/14 (92%)

Strand = Plus / Minus

Query: 148 gttcaaggaagcct 161

||||||| ||||||

Sbjct: 19 gttcaagaaagcct 6

Score = 21.1 bits (22), Expect = 6.4

Identities = 13/14 (92%)

Strand = Plus / Minus

Query: 148 gttcaaggaagcct 161

||||||| ||||||

Sbjct: 19 gttcaagaaagcct 6

Score = 21.1 bits (22), Expect = 6.4

Identities = 13/14 (92%)

Strand = Plus / Minus

Query: 488 atccgcgaggccga 501

|||| |||||||||

Sbjct: 14 atccacgaggccga 1

Score = 21.1 bits (22), Expect = 6.4

Identities = 14/16 (87%)

Strand = Plus / Plus

Query: 325 cctgatggctcgcaag 340

||||||| ||| ||||

Sbjct: 3 cctgatgcctcccaag 18

Score = 21.1 bits (22), Expect = 6.4

Identities = 13/14 (92%)

Strand = Plus / Minus

Query: 488 atccgcgaggccga 501

|||| |||||||||

Sbjct: 14 atccacgaggccga 1

Score = 21.1 bits (22), Expect = 6.4

Identities = 13/14 (92%)

Strand = Plus / Minus

Query: 148 gttcaaggaagcct 161

||||||| ||||||

Sbjct: 19 gttcaagaaagcct 6

>ptc-miR396g MIMAT0002037

Length = 21

Score = 21.1 bits (22), Expect = 6.4

Identities = 13/14 (92%)

Strand = Plus / Minus

Query: 147 agttcaaggaagcc 160

|||||||| |||||

Sbjct: 20 agttcaagaaagcc 7

>ptc-miR396f MIMAT0002036

Length = 21

Score = 21.1 bits (22), Expect = 6.4

Identities = 13/14 (92%)

Strand = Plus / Minus

Query: 147 agttcaaggaagcc 160

|||||||| |||||

Sbjct: 20 agttcaagaaagcc 7

>gma-miR1533 MIMAT0007396

Length = 19

Score = 21.1 bits (22), Expect = 6.4

Identities = 11/11 (100%)

Strand = Plus / Minus

Query: 591 attattatttt 601

|||||||||||

Sbjct: 17 attattatttt 7

>ahy-miR3510 MIMAT0016333

Length = 22

Score = 21.1 bits (22), Expect = 6.4

Identities = 11/11 (100%)

Strand = Plus / Minus

Query: 333 ctcgcaagatg 343

|||||||||||

Sbjct: 17 ctcgcaagatg 7

>aly-miR831 MIMAT0017614

Length = 22

Score = 21.1 bits (22), Expect = 6.4

Identities = 11/11 (100%)

Strand = Plus / Minus

Query: 527 tacgaggagtt 537

|||||||||||

Sbjct: 13 tacgaggagtt 3

>smo-miR1100 MIMAT0005256

Length = 22

Score = 21.1 bits (22), Expect = 6.4

Identities = 13/14 (92%)

Strand = Plus / Plus

Query: 233 ggacagaacccaac 246

||||||||||| ||

Sbjct: 7 ggacagaaccccac 20

>pab-miR396c MIMAT0018154

Length = 21

Score = 21.1 bits (22), Expect = 6.4

Identities = 13/14 (92%)

Strand = Plus / Minus

Query: 147 agttcaaggaagcc 160

|||||||| |||||

Sbjct: 20 agttcaagaaagcc 7

>pab-miR396b MIMAT0018153

Length = 21

Score = 21.1 bits (22), Expect = 6.4

Identities = 13/14 (92%)

Strand = Plus / Minus

Query: 147 agttcaaggaagcc 160

|||||||| |||||

Sbjct: 20 agttcaagaaagcc 7

Database: matureREL16.txt

Posted date: Oct 27, 2010 10:59 AM

Number of letters in database: 88,243

Number of sequences in database: 4142

Lambda K H

0.634 0.408 0.912

Gapped

Lambda K H

0.625 0.410 0.780

Matrix: blastn matrix:2 -3

Gap Penalties: Existence: 5, Extension: 2

Number of Sequences: 4142

Number of Hits to DB: 99,942

Number of extensions: 78011

Number of successful extensions: 78011

Number of sequences better than 10.0: 166

Number of HSP's gapped: 78011

Number of HSP's successfully gapped: 327

Length of database: 88,243

X1: 14 (12.8 bits)

X2: 33 (29.8 bits)

X3: 110 (99.2 bits)

S1: 14 (14.1 bits)

S2: 22 (21.1 bits)
