# Supplementary material for: On the complexity of miRNA-mediated regulation in plants: novel insights into the genomic organization of plant miRNAs
Source: Biol Direct. 2012 May 8;7:15. doi: 10.1186/1745-6150-7-15 (PMC3464803; doi:10.1186/1745-6150-7-15)
Supplement: Additional file 5 — Sequences of wheat SF-2 calmodulin genes (TaCaM2-1,TaCaM2-2,TaCaM2-3). Precursors, mature and reverse complement sequences are reported for miR1118 and miR1125. [file 1745-6150-7-15-S5.doc]

>gi|1754996|gb|U48690.1|TAU48690 Triticum aestivum calmodulin TaCaM2-1 mRNA, complete cds

CTTGATCTGTTTGCTTTGTCAGTATTGGTTAACGAGCGACCAGGAACTATCTGTTGGATCGTTCTCGGTT

TTGAGGGATCGCGGATCCCGATTCCTGGTCTGCACGCCTTCCTTTTGAATCCCCATCTATTGTGATTTCT

TGAGATCCGTTTGGAGTCTACGGACCTGCCGGCAATGCTTGACTGCTTGTCACTTTTGGATCTTAGAGAG

TGGCGTAGCTTGATTGGGGGTTGCATTACCACCAAGGAGCTGGGAACTGTCATGCGTTCCCTGGGGCAGA

ATCCCACTGAGGCAGAGCTTCAAGACATGATCAATGAGGTGGATGCTGACGGCAATGGAACAATTGATTT

CCCTGAATTCCTCAACCTTATGGCCCGCAAGATGAAGGACACTGATTCTGAGGAAGAACTCAAGGAGGCA

TTCCGTGTGTTTGACAAGGATCAAAATGGTTTTATCTCTGCTGCTGAACTGCGCCATGTCATGACCAACC

TTGGTGAGAAGTTGACTGATGAGGAGGTTGACGAGATGGTCCGTGAGGCTGATGTTGATGGTGATGGCCA

GATCAACTATGATGAATTTGTTAAAGTCATGATGGCCAAGTAAGTTGCATTACCACCTGAGGGAAGCTTC

AGTAATCAGGCCAAAATATTACCTATCCAGGTGCCATTTTGTGAGATGGCTATCGTATTAAACCGAAAAC

AATATAACACTGTGCTGTGTCAT**ACTCCCTCCGTTCCAAAATATAGTGCGCCCGCGCTTCCGGAGGTCCA**

**ACTTTGACCATAAATTTAACCAATGAGACCAACTGCGGCGGGAGAAAAAATTATATAATTGAAAACTTCT**

**TTCGAATACGAATTCACTGATATAATTTTTGCTTCCGCCGCAATCGGTCTTGGTAGTTAAATTTACGGTC**

**AAAGTTGAAGCACGTGGATAGAGGAAGCACTACATTATGGAATGGAGGGAGT**ATGTTGTAGGCAATGATT

GTTTGCCTTGAAGATTTTATAGATCCTGCTCTGGTCTTTCTGATTGTGTCTGTTCCTTTTTTTTATGTTT

GGTTCGTTTCAACTTTTCAAGAGTGTAGCCACAATGTGTTAATGAATAACTATATGTCAGTGTTCCATGA

TTGCA

>gi|1754998|gb|U48691.1|TAU48691 Triticum aestivum calmodulin TaCaM2-2 mRNA, complete cds

CGCATCGCCACCTTCCTCCCTCCTCCCCTCGCCATCTCCGACCCGACTTGCGGCCAATGGCGGACCAGCT

CACCGACGACCAGATCGCCGAATTCAAGGAGGCTTTCAGCCTCTTCGACAAGGATGGGGACGGTTGCATT

ACCACCAAGGAGCTGGGAACTGTCATGCGTTCCCTGGGGCAGAATCCCACTGAGGCAGAGCTTCAAGACA

TGATCAATGAGGTGGATGCTGACGGCAATGGAACAATTGATTTCCCTGAATTCCTCAACCTTATGGCCCG

CAAGATGAAGGACACTGATTCTGAGGAAGAACTCAAGGAGGCATTCCGTGTGTTTGACAAGGATCAAAAT

GGTTTTATCTCTGCTGCTGAACTGCGCCATGTCATGACCAACCTTGGTGAGAAGTTGACTGATGAGGAGG

TTGACGAGATGGTCCGTGAGGCTGATGTTGATGGTGATGGCCAGATCAACTATGATGAATTTGTTAAAGT

CATGATGGCCAAGTAAGTTGCATTACCACTTGAGGGAAGCTTCAGTAATCAGGCCAAAATATTACCTATC

CAGGTGCCATTTTGTGAGATGGCTATCGTATTAAACCGAAAACAATATAACACTGTGCTGTGTCAT**ACTC**

**CCTCCGTTCCAAAATATAGTGCGCCCGCGCTTCCGGAGGTCCAACTTTGACCATAAATTTAACCAATGAG**

**ACCAACTGCGGCGGGAGAAAAAATTATATAATTGAAAACTTCTTTCGAATACGAATTCACTGATATAATT**

**TTTGCTTCCGCCGCAATCGGTCTTGGTAGTTAAATTTACGGTCAAAGTTGAAGCACGTGGATAGAGGAAG**

**CACTACATTATGGAATGGAGGGAGT**ATGTTGTAGGCAATGATTGTTTGCCTTGAAGATTTTATAGATCCT

GCTCTGGTCTTTCTGATTGTGTCTGTTCCTTTTTTTTATGTTTGGTTCGTTTCAACTTTTCAAGAGTGTA

GCCACAATGTGTTAATGAATAACTATATGTCAGTGTTCCATGA

>gi|1755000|gb|U48692.1|TAU48692 Triticum aestivum calmodulin TaCaM2-3 mRNA, complete cds

AGAACACTCATTCGCATCGCCACCTTCCTCCCTCCCCCCCTCGCCATCTCCGACCCGACTTGCGGGCAAT

GGCGGACCAGCTCACCGACGACCAGATCGCCGAGTTCAAGGAGGCTTTCAGCCTCTTCGACAAGGATGGG

GACGGTTGCATTACCACCAAGGAGCTGGGAACTGTCATGCGTTCCCTGGGGCAGAATCCCACTGAGGCAG

AGCTTCAAGACATGATCAATGAGGTGGATGCTGACGGCAATGGAACAATTGATTTCCCTGAATTCCTCAA

CCTTATGGCCCGCAAGATGAAGGACACTGATTCTGAGGAAGAGCTCAAGGAGGCATTCCGTGTGTTTGAC

AAGGATCAAAATGGTTTTATCTCTGCTGCTGAACTGCGCCATGTCATGACCAACCTTGGTGAGAAGTTGA

CTGATGAGGAGGTTGACGAGATGGTCCGTGAGGCTGATGTTGATGGTGATGGCCAGATCAACTATGACGA

ATTTGTTAAAGTCATGATGGCCAAGTAAGTTGCATTACCACCTGAGGGAAGCTTGAGTAATCAGACCACA

ATATTACCTATCCAGGTGCCATTTTGTGGGATGGCTATTGTATTAAACCGAAAACAATATAACACTGTGC

TGTGTCAT*ATGTTGTAGGCAATGATTGTTTGCCTTGAAGATTTTATAGATCCTGCTCTGGTCTTTCTGAT

TTTGTCTGTTCCGTTTTTTTATGTTTGGTTCGTCAACTTTTCAAGAGTGTAGCCACAATCTGTTAATGAA

TAACTATATGTCAGTGTTCCATGATTACCCCTCAGTCATTGAAGTGTTATACTGTATGTGTGTCTGATAC

AGACATAGCTTGCATATTTTTTTGGTAACTATATAAATGAATATTTCTGGTAATGGAAGTGCGGTATCA

Bold characters: inserted sequence

Blue characters: miR1125 precursor

Highlighted in light blue, underlined: miR1125 mature sequence

Highlighted in light blue, not underlined: miR1125 reverse complement

Red characters: miR1118 precursor

Highlighted in yellow, underlined: miR1118 mature sequence

Highlighted in yellow, not underlined: miR1118 reverse complement

Highlighted in grey: miR118 target site

*= site of insertion
