# Supplementary figures and images for: On the complexity of miRNA-mediated regulation in plants: novel insights into the genomic organization of plant miRNAs
Source: Biol Direct. 2012 May 8;7:15. doi: 10.1186/1745-6150-7-15 (PMC3464803; doi:10.1186/1745-6150-7-15)

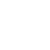

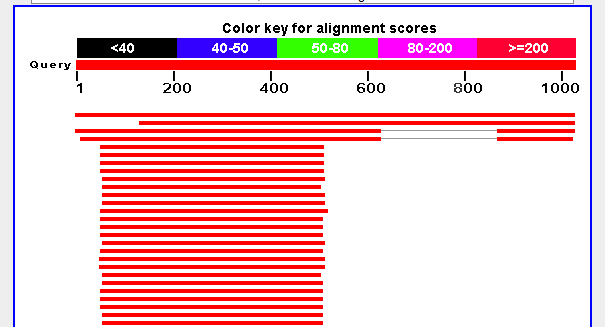

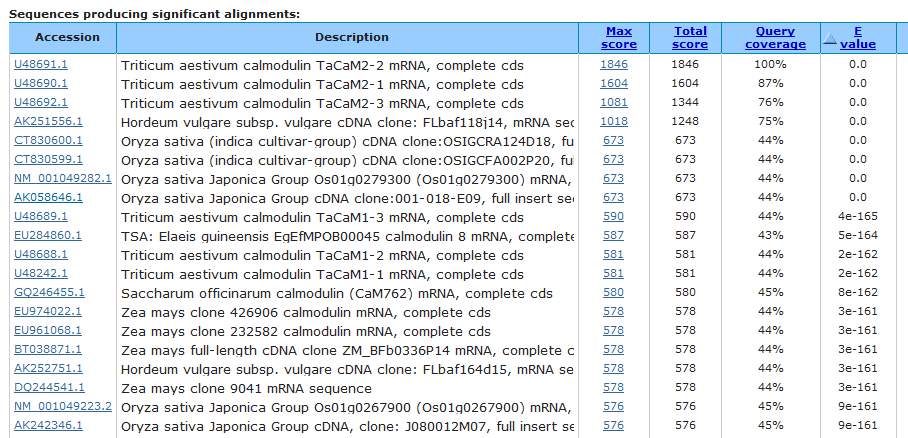

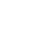

Supplement: Additional file 6 — Blast results related to the presence of the transposon-related insertion in calmodulin genes from plant species other than wheat using the sequence ofTaCaM2-2gene as query. [file 1745-6150-7-15-S6.doc]
