# Supplementary material for: On the complexity of miRNA-mediated regulation in plants: novel insights into the genomic organization of plant miRNAs
Source: Biol Direct. 2012 May 8;7:15. doi: 10.1186/1745-6150-7-15 (PMC3464803; doi:10.1186/1745-6150-7-15)
Supplement: Additional file 8 — Reports detailed on the adopted computational methods[15-17]. [file 1745-6150-7-15-S8.doc]

**Methods**

Plants gene indices (<http://compbio.dfci.harvard.edu/tgi/>) have been downloaded and blasted (e-value 0.001) against miRNA precursor sequences available on miRBase, on a species-specific basis ([http://mirbase.org](http://mirbase.org/), release 18). Only the sequences perfectly matching at least 95% of a miRNA precursor were retained for subsequent analysis. TC annotations have been recovered from fasta files downloaded from <ftp://occams.dfci.harvard.edu/pub/bio/tgi/data>.

The goal of the Gene Index Project is to use the available EST and gene sequences, along with the reference genomes wherever available, to provide an inventory of likely genes and their variants and to annotate these with information regarding the functional roles played by these genes and their products. Tentative Consensus sequences are thus created by assembling ESTs into virtual transcripts. In some cases, TCs contain full or partial cDNA sequences (ETs) obtained by classical methods. TCs contain information on the source library and abundance of ESTs and in many cases represent full-length transcripts. Alternative splice forms are built into separate TCs (<http://compbio.dfci.harvard.edu/tgi/definitions.html>).

MiRBase is the primary online repository for all miRNA sequences and annotation (Kozomara and Griffiths-Jones, 2011).

MiRNA target identification has been carried out with the psRNATarget tool with default parameters and the most recent Gene Index releases (Dai and Zhao, 2011) (<http://plantgrn.noble.org/psRNATarget>). The results were compared to the blast results in order to find sequences that code for a miRNA precursor, while having also one or more miRNA target sites.

The prediction of the secondary structure of the precursors and the Minimal Folding Free Energy Index (MFEI) were determined with the MFOLD 3.2 software (default parameters), freely available at <http://mfold.bioinfo.rpi.edu/cgi-bin/rna-form1.cgi> (Zuker, 2003).

MFEI = AMFE / (G+C)% where AMFE (Adjusted MFE) is the minimal free energy of 100 nucleotides.

The sequence alignments were performed with ClustalW on EBI server (<http://www.ebi.ac.uk/Tools/msa/clustalw2>) with default parameters.

Dai, X., and Zhao, P. X. (2011). psRNATarget: A Plant Small RNA Target Analysis Server. Nucleic Acids Res. Web Server Issue.

Kozomara, A., and Griffiths-Jones, S. (2011). miRBase: integrating microRNA annotation and deep-sequencing data. Nucleic Acids Res. 39, D152-D157.

Zuker, M. (2003). Mfold web server for nucleic acid folding and hybridization prediction. Nucleic Acids Res 31, 3406-3415.
