# Supplementary material for: On the complexity of miRNA-mediated regulation in plants: novel insights into the genomic organization of plant miRNAs
Source: Biol Direct. 2012 May 8;7:15. doi: 10.1186/1745-6150-7-15 (PMC3464803; doi:10.1186/1745-6150-7-15)

MIR1118 (miRBase precursor)

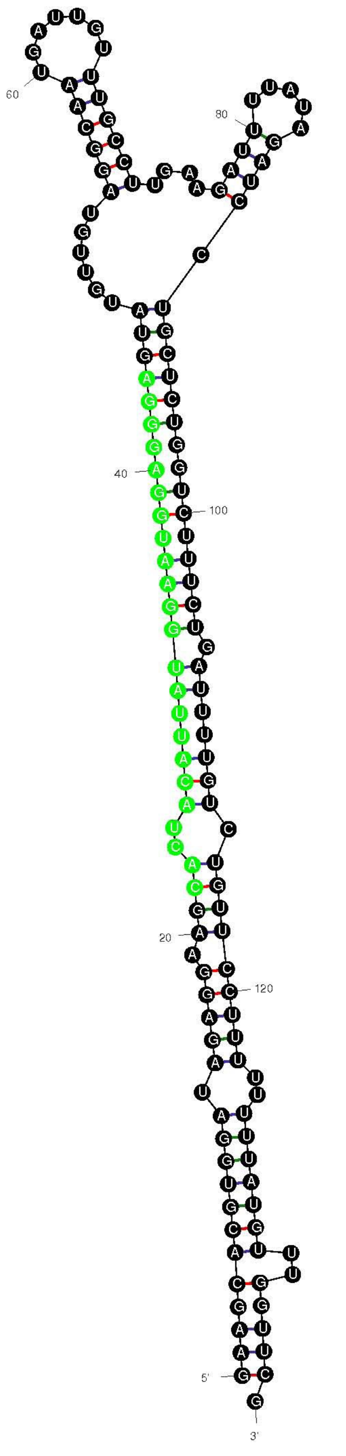

MIR1118 (putative precursor)

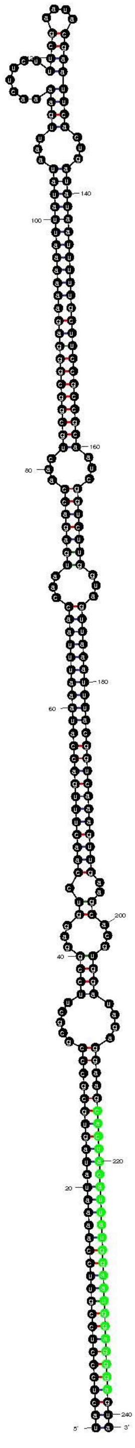

MIR1125 (putative precursor)

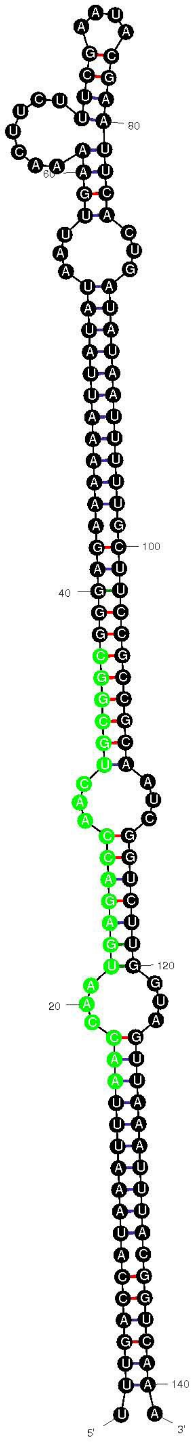

Supplement: Supplementary file 8 [file 1745-6150-7-15-S7.doc]
